# Unbiased Structure Prediction of Sophisticated Cage Structures

**DOI:** 10.1002/anie.9267462

**Published:** 2026-06-30

**Authors:** Andrew Tarzia, Giovanni M. Pavan

**Affiliations:** ^1^ Department of Applied Science and Technology Politecnico di Torino Torino Italy; ^2^ School of Chemistry University of Birmingham Edgbaston Birmingham UK

**Keywords:** cage compounds, computational chemistry, graphs, minimal models, structure prediction

## Abstract

Cage structure prediction has made significant strides by generating structures based on what the community has seen before. However, to computationally design and discover novel structures, the community must be able to evaluate and model all structural candidates. Here, we introduce unbiased structure prediction workflows in our software, *cgx*, facilitated by exploration algorithms and our low‐cost minimal models. By comparing to experiments, we show that our approach predicts cage structures starting only from the experimental inputs (building block types, features and their stoichiometry). We demonstrate the use of this method prior to any costly experimental commitment, providing an efficient automated approach that is open source and applicable to multiple model resolutions. By providing recipes with copyable code in the documentation, we make uptake of this new method as facile as possible for chemists with a wide‐range of expertise.

## Graphical abstract

We introduce the software and workflow for automated, unbiased exploration of all possible connectivities of a given set of building blocks and their stoichiometry to predict stable cage structures.

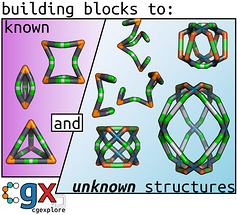

## Introduction

**FIGURE 1:**
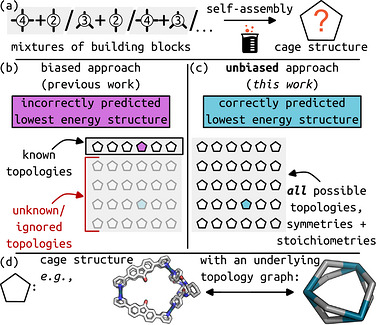
(a) The goal of structure prediction is to predict from a given mixture of building blocks, of any topicity, what structure will form. (b) Biased structure prediction approaches explore known topologies, incorrectly predicting the lowest energy structure. (c) Our new unbiased approach accesses the previously ignored and unknown topology, symmetry and stoichiometry space, finding a new lowest energy structure. (d) Example cage structure [16] and underlying topology graph.

In the field of porous and self‐assembled molecular cages [1], there has been significant effort to develop computational workflows for rationally designing or discovering new cages [2, 3, 4, 5]. The key challenge is to predict, prior to experiment, the self‐assembly outcome (Figure 1a), which is a process determined by, among other things, building block properties and experimental conditions. To do so, many structure generation approaches take a library of known topology graphs (that define how to connect building blocks to generate a cage model, Figure 1d) [6], and builds the associated library of cage structures from the building blocks of interest (Figure 1b) [7, 8, 9]. However, cages are incredibly diverse, a feature that underpins their tunability and promise towards many potential applications (e.g., catalysis [10], sensing [11], and separations [12, 13]), and the existing, biased approach to computational screening fundamentally limits design and discovery by only examining a prescribed initial set of cages. Additionally, cage diversity is growing with the advent and target of increasingly sophisticated, asymmetric structures, resulting in a more challenging modelling or prediction landscape. For example, using multi‐component building block mixtures (e.g., using two distinct ditopic molecules to form a heteroleptic cage) is a key approach to forming complex, multi‐functional cages [14, 15]. Our new unbiased approach (Figure 1c) allows the prediction and design of novel, asymmetric, and multi‐component structures by considering all possible structural and stoichiometric outcomes beyond those expected by previous works, effectively accessing structures that would previously have been ignored.

A clear example of when an unbiased approach was necessary arose in our recent work [16], where an unexpected stoichiometry of building blocks in a heteroleptic cage was discovered experimentally (Figure 1d). This work highlighted a disconnect between experimental and computational approaches, where the outcome could not be computationally rationalised or could not have been predicted without sufficient experimental data to suggest which prescribed topology and, in particular, stoichiometry to model. This fundamental barrier to computational methods hinders their wider application, specifically limiting their integration as predictive tools into experimental settings. In this previous work, we developed a stochastic structure prediction method that was able to correctly determine the most stable structure and stoichiometry starting only from experimental parameters, namely the potential stoichiometries and the building block structures.

The key challenge with the above approach was that many equivalent structures were generated, accentuating a persisting barrier to unbiased structure prediction, which is the number of structures that need to be explored. With significant numbers of structures, the cost of exploring a full landscape using atomistic representations, e.g., classical simulations, semiempirical methods or density functional theory (DFT), can become intractable. It may be possible to filter the number of studied structures based on geometric or established principles to save significant computation time, if said principles are known [17, 18]. Here, we have brought together a new graph‐based generation and filtering workflow, and our minimal model for cage structures [19] to achieve efficient unbiased structure prediction. We show that using low‐cost methods alongside high‐throughput screening or exploration algorithms is particularly critical for handling the combinatorial explosion of the search space when studying large, heteroleptic or asymmetric systems.

In this paper, we introduce new structure prediction algorithms with decreased barriers to usage through our open‐source Python package, *CGExplore* (“*cgx*”; https://github.com/andrewtarzia/CGExplore). In particular, our approach allows users to go directly from experimental input parameters (the building blocks of a reaction) to predicted structures. We demonstrate the use and modularity of *cgx* i) as an alternative approach to biased structure prediction workflows, opening up the possibility for novel cage discovery [20], and ii) as a platform for molecular systems design. This accessible framework is adaptable and will allow for structure explorations beyond expected stoichiometries and mixtures that were not straight‐forward to implement before, enabling rapid predictions of the self‐assembly outcomes of complex and sophisticated structures.

## 1 Performing Structure Prediction and Design With *cgx*

**FIGURE 2:**
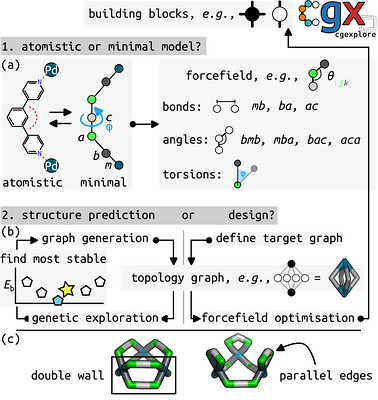
*cgx* flow chart, from building blocks through structure prediction or design. (a) Mapping between atomistic and minimal models that capture the key geometric features of the provided molecule, and an example definition of the minimal model forcefield components. (b) Structure prediction and design loops from user input, through defined topology graphs (shown in two dimensional and three dimensional representations) and either of the exploration or forcefield optimisation algorithms implemented in *cgx*. (c) Schematic of parallel edges and double walls on stoichiometry 3:6 structures.

We have developed an infrastructure to explore arbitrary cage models efficiently in *cgx* that is built on multiple modular algorithms and workflows. We show the application of *cgx* in structure prediction workflows, and, their inverse, building block design processes (Figure 2), where structure prediction aims to find the most stable structure(s) for a given set of building blocks (predicting self‐assembly outcomes) and building block design aims to determine which modifications can be made to the input building blocks to suggest new building blocks that stabilise the target structure or graph.

### 1.1 Defining the Ingredients of Cage Models

*cgx* allows users to predict accessible structures from a set of molecular building blocks and their stoichiometries. Here, we define the interface between computational methods and chemical design, and highlight the scope for exploration built into *cgx*. We have currently implemented three components of cage structure prediction (Figure 2): **building blocks**, **topology graphs** and **forcefields**. Diversity in cage design comes from the hierarchical nature of their structures, which we take advantage of in our structure prediction approaches that treat these three components as distinct features that can be optimisable variables [21].

The **building blocks** in this context are the distinct molecular precursors used in the self‐assembly process that are converted to models for cage construction. They are the main user input, and are parsed using the structure‐generation software, *stk* (https://github.com/lukasturcani/stk) [22], which defines the reactive functional groups used in construction. Importantly, cgx works with atomistic models, where the chemistry is included, and abstract, minimal or coarse‐grained (CG) models, where the key chemistry is considered and superfluous features are ignored (Figure 2a).

The **forcefield** defines the interactions in the theoretical model that represents a users building blocks/structures. The choice of the theoretical model depends on the use‐case of *cgx*: structure prediction of new systems (Section 2.2), system optimisation/design (Section 2.1) or high‐level predictions of a small number of candidates (Section 2.3). When using atomistic representations, the forcefield is defined for a given building block by the theoretical method used. In Section 2.3, we applied a semiempirical quantum mechanical method and DFT, neither of which apply a classical forcefield. Therefore, the **forcefield** is often not a variable, however, for many aspects of this work we use our minimal model forcefield [19] (see Section S3). The abstract nature of our minimal model facilitates efficient chemical space exploration (by, e.g., modifying specific geometric parameters, like the ‘binder angles’, Figure 2a, given by the *bac* term in our minimal model), using the forcefield as a variable. We show here three different use cases for different models to facilitate structure exploration or design, that are made accessible to users by the development of *cgx*.

To simplify the modelling of self‐assembled structures, *stk* [22] was developed to create molecular assemblies from smaller, distinct building blocks by performing placement and reactions. This process was achieved by using a graph representation of molecular assemblies, called a **topology graph**, where building blocks are placed on vertices of the graph, and are connected (reacted) along edges of the graph. In *stk*, the topology graph also defines where in Cartesian space the building blocks are placed, using known, idealised structures seen in the literature [6]. This approach is ideal for the structure prediction paradigm, where we apply mathematical graph tools to build/explore arbitrary *stk* topology graphs using *cgx*, covering all possible connectivities between a set of building blocks. To do so, we overcame challenges in initial vertex placement and how to determine if two graphs are equivalent (or isomorphic, see below) in *cgx*. We have developed the software interface to make filtering, defining and exploring the large graph space as accessible as possible.

### 1.2 The Approach to Structure Prediction and Topology Graph Generation

To predict the outcome of self‐assembly, we aim to determine the thermodynamically most stable structure, assuming that the experimental product will not be a kinetic species, or determined by experimental conditions/environment (solvent/ions/guests) that we do not model here. In cage workflows, it is common to use the relative total energy derived from either classical, semiempirical or DFT calculations as a metric for the most stable structure [2]. To apply this accurately across a diverse structure space, one must consider the effect of changing building block components or changing nuclearities/stoichiometries on the scaling of the energy with the number and type of interactions (e.g., bonds and angles). To do so, we can compare formation energies between structures. In this work, we compare the energy per number of building blocks in a cage structure, $E_{b}$, in all cases. This parameter was defined in our recent work [19] as an effective formation energy for our minimal model. However, for the atomistic models in Section 2.3, this is a rough approximation for normalising the energy as E is the total energy from semiempirical GFN2‐xTB or r2SCAN‐3c DFT calculations, not a formation energy.

The overall process for structure prediction is as follows:

1. Start from a set of building blocks, their forcefield and model.
2. Set the stoichiometry (s), e.g. two tetratopic metals and four ditopic ligands would be s=2:4. The difference between stoichiometries can often be defined by a multiplier, m, where s=2:4 has m=2 compared to s=1:2.
3. Generate viable topology graphs based on s with **cgx.scram.TopologyIterator**.
4. Explore these graphs through either brute‐force or exploration algorithms, such as a genetic algorithm (Section 2.2).
5. For each graph, build unoptimised models based on different algorithms for converting from graphs to three‐dimensional coordinates for the graph vertices.
6. Geometry optimise each unoptimised model for each graph to obtain cage models using some workflow. Currently, we use an automated workflow based on *OpenMM* for both minimal model and atomistic models [23]. These workflows are described in Sections S4–S6, and can be extended to semiempirical or DFT methods.
7. Extract the lowest energy structure for further analysis.

Within *cgx*, we provide the list of graphs for particular stoichiometries that we generated to avoid the cost of re‐generation for other users. Additionally, in practice, structure prediction requires evaluating as many possible stoichiometries as is reasonable (i.e., repeating from step 2 above) given the chemical nature of the building blocks. To generate the in‐built graph lists, we randomly created a number of topology graphs governed by the chemical rules prescribed by the building blocks, saving a graph to the list if it passes isomorphism checks against all other saved graphs. We used *rustworkx* [24] to check for isomorphism (*rustworkx* is a Python package for working with graphs and networks, it was much faster than the well known graph software *NetworkX* [25] for this task). The next step for us, to improve efficiency, is to use graph analysis to avoid wasted computations based on strained sub‐graphs, and to ensure symmetry is handled robustly. For example, our current algorithm cannot distinguish geometric isomers that have isomorphic graphs; i.e., *cis* and *trans* heteroleptic M_2_L_4_ isomers are isomorphic.

We initially sampled a maximum of $10^{4}$ graphs. We benchmarked our algorithm against the ability to find the experimentally observed graphs for s=1:2 stoichiometries with m=1−12, akin to the well‐known ${\mathrm{Pd}}_{x}L_{2x}$ metal‐organic cages by Fujita and co‐workers [26]. Only the s=12:24 system had close to $10^{4}$ non‐isomorphic graphs, highlighting the challenge with combinatorial explosion as the number of building blocks increases. Most importantly, our algorithm was able to generate the experimentally observed graph for all stoichiometries bar s=12:24. We then evaluated optional filters to remove graphs that were expected to be unstable for larger topologies, e.g., removing all graphs with “parallel edges” and “double‐wall connections”, where one building block is bound twice to the same building block (i.e., a bidentate configuration) and where two building blocks are connected by two (or more) other building blocks, respectively (Figure 2c). These constraints do exclude the well known M_2_L_4_, M_3_L_6_ and M_4_L_8_ structures. Doing so decreases the number of possible graphs (from the initial set that we generated) by ≈ 99% (Figure S3). In *cgx*, filtering options like these are available to the user to apply when appropriate, saving computations on unreasonable structures based on reapplying chemical intuition to unbiased structure prediction. We explored using this filtering and building more graphs (sampling up to $10^{5}$) without significant extra cost. Again, our generated set did not include the well known s=12:24 structure (Table S1). Importantly, due to the random nature of the graph generation, increasing the maximum number of samples even further is required to fully explore graphs with more constituents, where the balance of compute time and completeness is the root cause of the limitations above. We did not attempt to build any larger sets to overcome this issue with randomness in the graph generation, although we aim to resolve this in new releases of *cgx* by re‐evaluating graph‐generation algorithms.

## 2 Results and Discussion

We focus here on reproducing known cases to highlight how our method can be used in the future to provide confidence and guidance to experimental decision‐making. In particular, we highlight the challenges that arise in three case studies based on conventional approaches, and how the utilities in *cgx* resolve those challenges. In this work, we have mapped atomistic systems to our minimal model [19] based on manual determination of key geometrical features, their bond lengths and angles (Section S9). Using the same model with the new exploration algorithms, we successfully reproduced our recently published work with improved efficiency (Section S10) [16]. Coupling minimal models with our workflows is very efficient (building a cage in seconds on standard hardware) for performing structure prediction and design. In the final example, we also show the use of atomistic models in unbiased structure prediction, highlighting the versatility of our approach.

### 2.1 Predicting the Structure of Ditopic + Tritopic Porous Cages and Exploring Forcefield Modifications

An important aspect of experimental decision‐making is how to find new building blocks or modify known building blocks to favour a specific structural target. In this case study, we couple structure prediction with parameter optimisation to explore using *cgx* for system design. In particular, we examine the role of flexibility in Zirconium–carboxylate cages (m=1,2 with s=2:3, building blocks shown in Figure S7) studied in Ref.  [27], where either **TwoPlusThree** or **FourPlusSix** cages [27], or mixtures of these (cage topology terminology aligns with experimentally known graphs when possible; Table S1) were observed.

**FIGURE 3:**
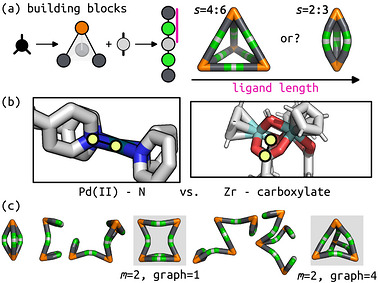
(a) Summary of the ditopic and tritopic building blocks used in the first case study, examining the role of ditopic building block length. (b) Schematic showing the changes in “complexity” when mapping to our minimal model a Pd(II)‐pyridyl reaction compared to Zr–carboxylate bond formation. Yellow circles highlight the connection points used in minimal model representations. (c) Graphs generated for m=1,2 and s=2:3, highlighting the stable graphs found in this case study.

We performed structure prediction for tritopic building blocks combined with a series of ditopic building blocks (Figures 3a and S7). Studying the zirconium–carboxylate‐based cages, we aim to recapture the stability of the smaller s=2:3 topology across ligand lengths (ligand width is also considered in the published work, but we do not model it here) [27]. We show that an s=4:6 cage is the lowest energy graph in all cases (Figure S7). In particular, either graphs 1 (the known **FourPlusSix2** topology graph; Table S1) [28] or 4 (the expected topology) for s=4:6 (Figure 3c) are the preferred structures. We also do not predict the preference for s=2:3, **TwoPlusThree** structures in any case, disagreeing with the observed phase diagram in Ref.  [27].

We hypothesised that flexibility and a non‐trivial mapping of “hinge” points in the atomistic building blocks introduces challenges to parameterisation of our minimal model (compare the linear Pd–pyridyl interactions we recently focussed on [19] to the multi‐connected zirconium–carboxylate bonding with respect to geometrical simplicity, Figure 3b). This highlights a challenge with the application of minimal models based on only the building block geometries, where significant deviations/flexibility from the input geometries to those achieved in the cages will limit the predictability of the method. We overcome this challenge in *cgx* by providing a structure design workflow based on forcefield parameter optimisation.

**FIGURE 4:**
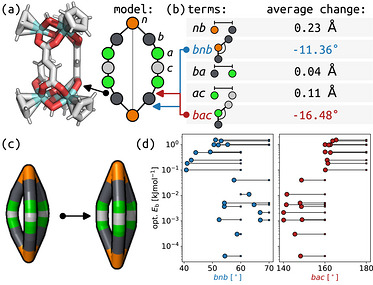
(a) Example **TwoPlusThree** structure (REFCODE: LUSQIC) [27] compared to a two dimensional representation of the minimal model of the equivalent structure, highlighting bead types. (b) Forcefield terms modified in optimisation process, with their average change across structures (including all models and forcefields used in the initial screen), highlighting the two impactful angles, and mapping that to structural effects in (a). (c) Example of structures before and after forcefield optimisation. (d) Effect of parameter optimisation on two chosen terms, and the minimum achieved $E_{b}$ for **TwoPlusThree** structures. Point colours in (d) match the schematic in (b). The data for all other terms is shown in Figure S9.

**Parameter optimisation for system design**. Benefiting from the abstract nature of the minimal forcefield, we initially manually changed the input parameters (e.g., introducing a bend in the ditopic building block by changing the *bac* angle of 180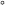 to 160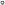, Figure 4b) and found an improved stability of the s=2:3 structure, highlighting the importance of flexibility (Section S12). However, human intervention brings bias and slows implementation. Therefore, we implemented continuous parameter optimisation of the input forcefield parameters to answer the question: “how far do we have to change the forcefield to favour the s=2:3 structure?” This process (Figure 4c) takes a specific structure and performs a “dual annealing” optimisation (using *scipy* [29]) over selected forcefield terms to minimise $E_{b}$ (Section S11).

We optimised over the selected bond and angle terms (*ba*, *bac*, *nb*, *bnb* and *ac*; Figure 4b) from the initial values and found that, on average, decreases in $E_{b}$ are achieved by improving the matching of the angles between the tritopic and ditopic building blocks (*bnb* and *bac*). These angles are the pyramid angle of the tritopic building block and the binder angle of the ditopic building block, respectively. Due to the maximum change we allowed in the optimisation algorithm, very low $E_{b}$ values are only achieved when starting from already bent (*bac* = 160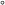) ditopic building blocks. This general trend agrees with the experimental outcomes, where the **TwoPlusThree** structure exhibits a significant bend at the carboxylate–zirconium interaction (Figure 4a). Such an effect is expected to depend on length, flexibility and other interactions not included in our model, including the steric effects used in Ref.  [27].

This new tool, built into *cgx*, allows the user to learn from an initial structure prediction result with our minimal model and perform building block design. By starting from an initial forcefield, defined by the users building blocks, a target structure (which could be an experimentally relevant target) may be deemed unstable, with a high strain energy. Now, the user can explore forcefield changes that stabilise their target topology graph or structure, providing principles to design their next building blocks. Our future works will aim to automate the conversion between atomistic and minimal models to simplify this design process.

### 2.2 Applying Our Method to Higher Nuclearity Heteroleptic Cu_18_L^*A*^L^*B*^ Cages

**FIGURE 5:**
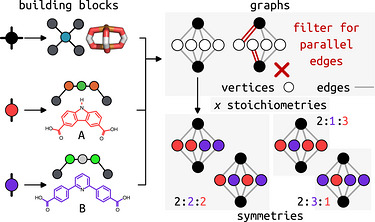
Heteroleptic building block mixture from Ref.  [33] and the symmetry differentiation over explored graphs (removing graphs with parallel edges, shown by the red cross) and stoichiometries when studying heteroleptic systems.

The design of high nuclearity, asymmetric cages is of significant interest to the field, but is very challenging due to the number of possible structures that need to be explored [17, 18]. For heteroleptic or unsymmetrical mixtures of building blocks, structure prediction must explore all topology graphs, as above, but also the configuration (or symmetry) of building blocks on that graph, which is also a function of the stoichiometry of the mixture (Figure 5), leading to a significant increase in degrees of freedom. Additionally, subtle effects, such as external functionalisation, ligand width and ligand size ratios have been shown to alter isomer preferences among these large structural spaces [30, 31, 32]. We couple *cgx* with our minimal model and exploration algorithms (here, a genetic algorithm) to overcome the challenge of combinatorial explosion and predict the self‐assembled structure and stoichiometry of a 27‐unit, mixed‐ligand metal–organic cage, significantly decreasing the number of necessary calculations and making unbiased structure prediction of large structures tractable on standard hardware.

**FIGURE 6:**
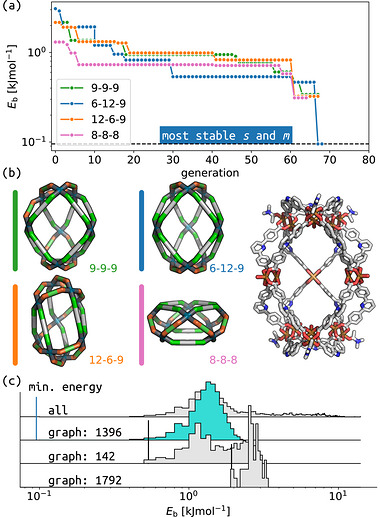
(a) Progress of the hierarchical GA run towards the most stable structures for larger m∗s mixtures. (b) Most stable structures from the runs in (a), where the blue structure is the most stable overall and the experimental outcome with m∗s=6:12:9. (right) Crystal structure obtained from Ref.  [33] (REFCODE: QUHSES). (c) $E_{b}$ distributions of building block configurations in all graphs or the three most “elite” graphs for m=1 and s=6:12:9, labelled by their graph index, or identifier. The experimental graph index distribution is shown in cyan.

Li et al. generated two heteroleptic Cu_18_ (treated here as M_9_ square‐planar) cages from copper paddlewheel structures (crystal structure in Figure 6b) [33]. Self‐sorting of this 27‐component system to one species was confirmed by x‐ray diffraction only and experimental conditions were shown to have an impact on the outcome, highlighting the other factors in structure prediction. We focus only on one of their building block pairs, based on the symmetrical building blocks in Figure 5 (Section S15), although handling asymmetric building blocks with our approach is possible and currently under‐development. We initially ran this case study in the same way as those above: defining possible graphs and building block configurations, then building all of them. However, the number of possible structures was very large, and we hypothesised that many structures would be wasted effort. Therefore, we introduced a multi‐step genetic algorithm (GA)‐based workflow (Section S14) that finds top scoring graphs and building block configurations in less computations than brute‐force searching. To test this, we scanned m=1–9 with s=1:1:1, 2:4:3 or 4:2:3, where the ratios are associated with building blocks as L^*A*^:L^*B*^:M and a maximum M=9 was allowed. The experimental structure (Figure 6b has 9 paddlewheel building blocks, 6 ditopic ligand A and 12 ditopic ligand B, giving a formula M_9_L^*A*^_6_L^*B*^_12_ with s=6:12:9. We mapped the two building blocks based on the given angles of 90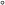 and 120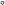 for A and B, respectively.

For small m, we quickly explore the whole landscape and this GA‐based approach is unnecessary. An initial GA, not shown, highlighted that including parallel edges (Figure 5) was not likely to find high fitness candidates. Hence, initial screening can help rule out large areas of structure space, which we applied here. The GA‐based approach explores the graph and building block configuration landscape in short initial runs, leading to an “elite” graph index (Figure 6c), which can be focussed on in the second and third steps, effectively exploring all building block configurations for those elite graphs. Figure 6a shows that the s=6:12:9 stoichiometry structure is indeed the lowest in energy of those studied. Ultimately, many thousands of structures were enumerated, but we predicted the experimental outcome in a few days on standard computational hardware (13th Gen Intel i9‐13900 (32) CPU with NVIDIA GeForce RTX 4070 GPU). Additionally, changing the input parameters by small amounts does not alter the predicted outcome (Section S15).

Based on this case study, we have shown how *cgx* allows users to explore efficiently and without bias across stoichiometries and graphs for mixed‐component systems with minimal human intervention. To further highlight the adaptability of our approach to mixed systems, we show in Section S13 how to explore heteroleptic cages from four components [34]. Prior to these developments, such predictions were not accessible to experimental chemists without initial experimental data to narrow down the structure space.

### 2.3 Performing Structure Prediction at the Atomistic Scale

**FIGURE 7:**
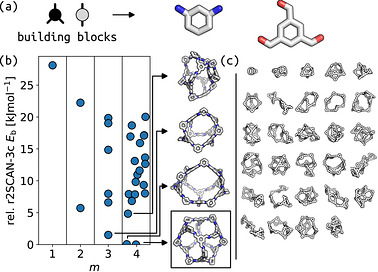
(a) Building block mixture explored. (b) Relative r2SCAN‐3c energies of atomistic structures with m=1,2,3,4 and s=2:3. For m=4, a random number is used to displace data on the *x*‐axis, to highlight overlapping points. The structures within $5\;kJ\;{\mathrm{mol}}^{-1}$ are shown. (c) All graphs evaluated.

Our approach is built on *stk* [22] and model/chemistry‐agnostic graph representations of constructed molecules. Therefore, it transfers across model resolutions. To bring our new approaches closer to the experimental setting, we wanted to ensure that it applied to atomistic models, where the user does not need to define a minimal model. In particular, we examined how to use unbiased structure prediction in a high‐throughput screening workflow, filtering from a large number of graphs with lower‐cost methods to a high‐cost ranking of top‐candidate structures. We performed structure prediction with atomistic models using a cheap geometry‐optimisation workflow (Section S6) of a large porous organic **EightPlusTwelve** cage structure formed from the imine condensation of the building blocks in Figure 7a (these are unfunctionalised equivalents of the building blocks used in Ref.  [35]). We explored m=1,2,3,4 and s=2:3 (Section S7), removing any graphs with parallel edges.

Figure 7 shows all graphs explored (29 in total) alongside the predicted structures within $5\;kJ\;{\mathrm{mol}}^{-1}$ of the lowest energy structure. The structures were geometry optimised with the Sage 2.2.1‐unconstrained forcefield [36] in *OpenMM* [23] using the MMFF94 charge method, then with GFN2‐xTB [37], and their energies were computed using GFN2‐xTB and compared to the composite DFT method, r2SCAN‐3c [38] (Section S6). The experimental cage is the most stable by < $1\;kJ\;{\mathrm{mol}}^{-1}$ when comparing the r2SCAN‐3c energies (Figure S1 shows a similar ranking is achieved with the GFN2‐xTB energies). This workflow does not confidently distinguish between four structures within $5\;kJ\;{\mathrm{mol}}^{-1}$. We expected that using a higher level of theory, alongside DFT‐based geometry optimisation [6] may be necessary to confidently predict atomistic outcomes. Hence, structures within $5\;kJ\;{\mathrm{mol}}^{-1}$ of the lowest energy based on the r2SCAN‐3c energies were further optimised with r2SCAN‐3c to examine if DFT‐level geometry optimisation would improve the predictions, or differ from lower‐cost methods. Computing $E_{b}$ after r2SCAN‐3c geometry optimisation of the top four candidates did not alter their close relative energies, with the experimental outcome still < $1\;kJ\;{\mathrm{mol}}^{-1}$ more stable than the next most stable structure.

This case study highlights how to use high‐throughput screening and unbiased structure prediction on atomistic models. There remain challenges associated with the stereochemistry of the cyclohexane building blocks and the cages due to the orientation of the imines emanating from the benzene rings (Section S7), which can be overcome through judicial choice of building block models. Overall, for the cost of an overnight calculation (without the DFT calculations) on standard hardware, this workflow narrows the structure space significantly (initially from 29 down to 4 candidate structures) to aid in experimental design or decision making, clearly supporting the formation of larger cages, rather than the archetypical **FourPlusSix** structure (m=2).

## Conclusion

We have introduced a series of tools built into our open‐source software, *cgx* (https://github.com/andrewtarzia/CGExplore), for performing unbiased structure prediction to connect experimental building blocks with their most stable possible self‐assembly products. While we did not predict any new structures in this paper, we highlighted in three case studies the key ways that unbiased structure prediction resolve limitations of the conventional approach, which are implemented through a simple interface in *cgx*. For example, we show the use of a genetic algorithm to overcome the combinatorial explosion in structure prediction of complex molecular systems. Coupled with the forcefield optimisation in Section 2.1, *cgx* allows users to learn how to design new building blocks. *cgx* also allows high‐throughput screening of possible structures with minimal user input at the atomistic level.

This paper focusses on the prediction of heteroleptic cage structures. While the methods can be applied to other asymmetrical systems in the future, this is already a significant step in the toolbox of structure prediction methods in the field of cage self‐assembly. The current work built on our minimal model is best suited to larger, rigid structures, where geometry plays a key role in self‐assembly outcome. It is important to note that this model does not account for key factors that can determine the self‐assembly outcome experimentally, including entropic and solvent/counter‐ion effects (e.g., templation) [9, 39, 40]. For example, using minimal models to predict *cis*‐M_2_L^*a*^_2_L^*b*^_2_ Pd(II) cages (reproducing our work in Ref.  [8]), was unsuccessful (data not shown). However, the multi‐scale nature (applied also to atomistic models) and provided exploration algorithms alleviate these concerns for the wide utility of *cgx* because they allow users to apply appropriate models/approaches considering their cost‐benefit analysis.

These developments upend the conventional high‐throughput computational screening workflow, where the potential structures/topologies are chosen by a human (a process that is inherently biased), to now suggest structures purely based on the building block inputs. Removing bias is crucial for predicting novel and interesting materials. We have engrained user‐friendliness into *cgx* with robust documentation and downloadable, pre‐compiled recipes (https://cgexplore.readthedocs.io/en/latest/recipes.html) that reproduce many of the examples shown here. Additionally, we provide an interactive visualisation [41] of the structures generated in this work, and others, at https://cgmodels.readthedocs.io/en/latest/ to allow users to examine the possible cage landscape. This ease‐of‐use will inspire experimental chemists to interact with this workflow and explore ligand combinations in previously unexplored regions, while the modular software leaves space for future development. In particular, the chemistry agnostic algorithms allow unbiased structure prediction to be applied beyond self‐assembling cages.

Publications cited in the supporting information are included here [6, 16, 17, 19, 21, 22, 23, 24, 25, 27, 28, 29, 33, 34, 35, 36, 37, 38, 41, 42, 43, 44, 45, 46, 47, 48, 49, 50, 51, 52, 53, 54, 55].

## Author Contributions

**Andrew Tarzia**: conceptualisation, data curation, formal analysis, funding acquisition, methodology, software, visualisation, writing – original draft, reviewing and editing. **Giovanni M. Pavan**: writing – reviewing and editing, funding acquisition.

## Conflicts of Interest

There are no conflicts of interest to declare.

## Supporting information




**Supporting File 1**: anie73048‐sup‐0001‐SuppMat.pdf.

## Data Availability

The code for structure generation and analysis can be found at https://github.com/andrewtarzia/topology_scrambler and on Zenodo at https://doi.org/10.5281/zenodo.20034694. Data for this paper, including structure files and properties, are available at Zenodo at https://doi.org/10.5281/zenodo.20034390. A selection of structures are available for visualisation on our website: https://cgmodels.readthedocs.io/en/latest/.
