# Supplementary material for: Unbiased Structure Prediction of Sophisticated Cage Structures
Source: Angew Chem Int Ed Engl. 2026 Jun 30;65(34):e9267462. doi: 10.1002/anie.9267462 (PMC13480674; doi:10.1002/anie.9267462)
Supplement: Supplementary file 1 [file ANIE-65-e9267462-s001.pdf]

Supplementary Information for

# Unbiased structure prediction of sophisticated cage structures

**Andrew Tarzia** [a,b] **and Giovanni M. Pavan** [a]

* *E-Mail: a.tarzia@bham.ac.uk*

## S1. Software

A Python 3.11 environment is defined in the source code in the "environment.yml" file.

- atomlite (https://github.com/lukasturcani/atomlite): 4.4.5
- bbprepared (https://github.com/andrewtarzia/bbprepared): 2024.12.15.2
- cgexplore (https://github.com/andrewtarzia/CGExplore): 2025.2.5.1
- chemiscope[S1]: 0.8.3
- NetworkX[S2]: 3.6.1
- numpy[S3]: 1.26.4
- openff-toolkit: 0.18.0
- openforcefields: 2026.1.0
- openmm:[S4] 8.2.0
- openmmtools (https://github.com/choderalab/openmmtools): 0.24.1
- RDKit[S5]: 2024.3.4
- rmsd[S6,S7]: 1.6.4
- rustworkx[S8]: 0.16.0
- scipy[S9]: 1.15.1
- stk[S10]: 2024.10.2.0
- stko (https://github.com/JelfsMaterialsGroup/stko): 2025.12.7.1
- xtb[S11]: 6.7.1
- CREST[S12]: 3.0.1

## S2. Topology definitions

Alongside the new topology graph generation algorithms, we have used known topology graphs following the naming convention (Table S1) described in Ref. S13.

[a]Department of Applied Science and Technology, Politecnico di Torino, Corso Duca degli Abruzzi 24, 10129 Torino, Italy
[b]School of Chemistry, University of Birmingham, Edgbaston, Birmingham, B15 2TT UK

**Table S1:** Definition of known topologies studied in this work and the number of constituent building blocks used in $E_\mathrm{b}$ calculation. * suggests a modification on the named *stk* class was performed or that the topology was defined from scratch for this paper; these definitions are in *cgx*.

| topology | *stk* [S10] class | num. building blocks |
|---|---|---|
| **Tri$^2$Di$^3$** | TwoPlusThree | 5 |
| **Tri$^4$Di$^6$** | FourPlusSix | 10 |
| **Tri$^4_2$Di$^6$** | FourPlusSix2 | 10 |
| **Tri$^6$Di$^9$** | SixPlusNine | 15 |
| **Tri$^8$Di$^{12}$** | EightPlusTwelve | 20 |
| **Tet$^2$Di$^4$** | M2L4Lantern | 6 |
| **Tet$^3_3$Di$^6$** | M3L6 | 9 |
| **Tet$^4_4$Di$^8$** | M4L8* | 12 |
| **Tet$^4_2$Di$^8$** | -* | 12 |
| **Tet$^5_5$Di$^{10}$** | FivePlusTen | 15 |
| **Tet$^6$Di$^{12}$** | M6L12Cube | 18 |
| **Tet$^6_3$Di$^{12}$** | -* | 18 |
| **Tet$^8$Di$^{16}$** | EightPlusSixteen | 24 |
| **Tet$^8_4$Di$^{16}$** | -* | 24 |
| **Tet$^{10}$Di$^{20}$** | TenPlusTwenty | 30 |
| **Tet$^{12}$Di$^{24}$** | M12L24* | 36 |

## S3. Minimal model forcefield

The forcefield used for each minimal model [S14] is based on bond length, bond angle, torsional angle and excluded volume terms, and has a potential energy form

$$E = E_\mathrm{bond} + E_\mathrm{angle} + E_\mathrm{torsion} + E_\mathrm{excl.vol.}, \quad \text{[S1]}$$

where the functional forms of each term are

$$E_\mathrm{bond} = \frac{1}{2} k_\mathrm{bond} \left(r - r_0\right)^2 \quad \text{[S2]}$$

$$E_\mathrm{angle} = \frac{1}{2} k_\mathrm{angle} \left(\theta - \theta_0\right)^2 \quad \text{[S3]}$$

$$E_\mathrm{torsion} = k_\mathrm{torsion} \left(1 + \cos\left(p\phi - \phi_0\right)\right) \quad \text{[S4]}$$

$$E_\mathrm{excl.vol.} = \sqrt{\epsilon_i \epsilon_j} \left(\frac{\sigma_i + \sigma_j}{2r}\right)^{12}, \quad \text{[S5]}$$

where $r$ is the bond length (or distance between two beads), $r_0$ is the equilibrium bond length, $\theta$ is the angle, $\theta_0$ is the equilibrium angle, $\phi$ is the torsion, $\phi_0$ is the equilibrium phase offset, $p = 1$, $\epsilon_i$ is the strength of nonbonded interaction for bead $i$ and $\sigma_i$ is the size of bead $i$. $E_\mathrm{excl.vol.}$ is a force term defined such that there is a penalty for bead overlap. When beads are connected by two or less bonds, the $E_\mathrm{excl.vol.}$ term is turned off. We have selected parameters such that the bond, angle and torsion terms are on the same scale as all-atom molecular force fields (when present) with $k_\mathrm{bond} = 1 \times 10^5$ kJ/(mol nm$^2$), $k_\mathrm{angle} = 1 \times 10^2$ kJ/(mol rad$^2$), $k_\mathrm{torsion} = 50$ kJ mol$^{-1}$. We have used the same $\epsilon_i = 10$ kJ mol$^{-1}$ and $\sigma_i = 1$ Å for all beads. For four connected beads, *i.e.*, those mimicking square-planar Pd(II), the equilibrium angle is set to 90° between adjacent beads and 180° for opposite beads. This definition removes the possibility of rearrangements of the bonding pattern on these building blocks. We have implemented artificial torsions to mimic the rigidity of building blocks by aligning regions of the molecule, or enforcing planarity; all other torsions are ignored.

## S4. Geometry optimisation sequences

In structure-prediction workflows, the geometry optimisation algorithm or workflow is often designed to find the lowest energy conformation given the model parameters and be as efficient as possible, while maintaining modularity, transferability and accuracy. As this work tackled a series of different case studies (at multiple model resolutions), we have implemented a variety of different workflows built from a variety of modular steps (detail in Sections S5 and S6). This work was also hindered by the new challenge of optimising from arbitrary cage graphs, no longer benefiting from the initial geometrical guesses in well-defined *stk* graphs (designed to match to experimental structures; Section 1.1). [S10] Hence, we have used the graph-based software *NetworkX* (a Python package for the study of graph networks) [S2] to generate multiple reasonable initial coordinates for each graph, which are used in a sequential geometry optimisation workflow:

1. Define cage topology graph, *i.e.*, the connectivity.
2. Set topology graph vertex positions (initial coordinates) by generating vertex positions:
    - on a sphere, with no alignment algorithm
    - using *NetworkX* [S2] layout algorithms ("spring", "kamada", "spectral") based on the topology graph connectivity
    - using *NetworkX* [S2] layout algorithms ("spring", "kamada", "spectral") based on the bead/atom positions after initial construction of the cage
3. Perform geometry optimisation workflow.

With modular solutions to each step of that process, we show applicability to a wide range of challenging systems. While a complete benchmarking is needed, Figure S4(a) shows the cost of an optimisation sequence to be approximately 10s of seconds, even for the largest structures. In particular, the graph layout options from *NetworkX* were crucial to converting an arbitrary graph into three-dimensional coordinates without building block overlap and bond cross over. Figure S4(b) shows the count of the minimum energy structures achieved from specific steps in the geometry optimisation workflow, where *NetworkX* steps result in the majority (note that this data is from a benchmark run not reported on in this work). To further avoid overlap, we tested each layout with multiple scaling factors to increase the distance between building blocks. Using multiple initial configurations helps minimise the risk that one workflow fails, although it increases computational expense. Molecular dynamics (MD) and geometry optimisations of minimal model and atomistic systems are completed with free software, such as *OpenMM* (with the Sage 2.2.1-unconstrained[S15] forcefield),[S4] RDKit,[S5] and xTB.[S11] After optimisation, we extract the energy per number of building blocks in a cage ($E_{\mathrm{b}}$) as effective formation energies to compare across stoichiometries and nuclearities.

## S5. Minimal-model geometry optimisation sequences

The lowest energy conformer accessed in this process is saved as the final conformer of the model. Depending on the case study, a different sequence of steps (**A**, **B**, **C** or **D**; Table S2) was used. However, the components within those workflows were mostly constant:
**Assign**: Terms were assigned based on the structure coordinates and input forcefield.
**Constrained optimisation**: A geometry optimisation was performed for 20 steps, where all bond and angle potential terms were softened by a factor of 10, all torsions were switched off, and constraints were applied to all atoms not part of bonds formed by *stk*. This is akin to the constrained geometry optimisations applied in atomistic systems.[S16]
**Geometry optimisation**: A geometry optimisation was performed with the full force field applied and no constraints.
**Shifting**: A series of model conformers were generated and then geometry optimised, where all instances of a single bead type were displaced away from the model's centroid by 1, 2, 3 or 4 Å. This was repeated for each bead type.
**Neighbours**: Geometry optimisations were performed starting from pre-optimised cage structures from other attempts (with different initial conditions). In **A**: The forcefield of the current target was reassigned based on the pre-optimised structure. In **B**, it was not.
**Graphs**: New initial structures were generated from the *stko*-*NetworkX* graph of the atoms/beads and their connectivity using in **C**: spectral, random, spring and kamada kawai layouts and **D**: spectral and kamada kawai layouts. Each layout was also tested with a position scale multiplier of 5, 10 or 15. The forcefield was reassigned for each structure and then optimised with the full forcefield.
**Soft MD**: A MD simulation was performed starting from the lowest energy conformer accessed so far, where the bond and angle terms were softened by a factor of 10 and all torsions were switched off, allowing for conformational space exploration. The simulation was performed in the NVT ensemble with Langevin dynamics for 20000 steps (collecting a conformer every 500 steps, 40 in total) with a friction coefficient of 1 ps$^{-1}$ and a time step of 0.5 fs at 300 K. All bead masses were set to 10 amu, which are small for the average building block used in realistic cage systems. Every conformer was then geometry optimised with the full force field parameters.
**Random**: Ten random position matrices were tested with no forcefield reassignment.

**Table S2:** Geometry optimisation sequences applied to minimal models.

| step | A | B | C | D |
|---|---|---|---|---|
| **1** | Assign | Assign | Assign | Assign |
| **2** | Constrained optimisation | Constrained optimisation | Constrained optimisation | Constrained optimisation |
| **3** | Geometry optimisation | Geometry optimisation | Geometry optimisation | Geometry optimisation |
| **4** | Shifting | Shifting | Shifting | Graphs |
| **5** | Neighbours | Neighbours | Graphs | Random |
| **6** | Soft MD | Soft MD | Random | - |

## S6. Atomistic geometry optimisation sequence

Building blocks were selected as the lowest RDKit/Merck Molecular Forcefield (MMFF)[S17] energy in an RDKit/ETKDGv3[S18,S19] ensemble built with *bbprepared* (https://github.com/andrewtarzia/bbprepared). Automated cage construction was completed with the same process as minimal models, but a geometry optimisation sequence based on atomistic models was applied:

1. An MCHammer (https://github.com/andrewtarzia/MCHammer) optimisation was performed with a target bond length of 2 Å. MCHammer uses a rigid-body Monte-Carlo algorithm to bring the building blocks of a structure together by minimising the bond lengths of the connections created during *stk* construction.

2. Two subsequent geometry optimisations with MMFF[S17] in *stko* were performed.

3. Geometry optimisation using *OpenMM*[S4] was performed, where all bonds not modified during *stk* construction were restricted. We used the Sage 2.2.1-unconstrained ("openff_unconstrained-2.2.1")[S15] forcefield from *openff-forcefields* (https://github.com/openforcefield/openff-forcefields) and the MMFF94 charge method.

4. An unrestricted geometry optimisation was completed with Sage 2.2.1-unconstrained.

5. A conformer search was performed starting from the *OpenMM*-optimised cage structure using MD with Sage 2.2.1-unconstrained at 300 K, with the Langevin integrator using a friction coefficient of 10 $ps^{-1}$ and time step of 0.5 fs. A short equilibration was run for 10 ps and the production run performed for 200 ps. From the production run, 50 conformers were extracted and geometry optimised with *OpenMM*.

6. The lowest energy conformer was then geometry optimised with GFN2-xTB[S11] with normal convergence criteria (energy converged within 5E-6 Hartrees and gradient norm converged within 1E-3 Hartree bohr-1). The energy of this structure was saved, and divided by the number of building blocks in the structure to give $E_{\mathrm{B}}$.

## S7. Data for "Performing unbiased structure prediction at the atomistic scale."

This case study can be run using the script: "`atomistic_systems.py`". The goal of this study was to predict an isostructural model of the imine-based cage termed "CC20".[S20] In that study, the authors used a trialdehyde building block with a single alcohol group functionalised on the benzene ring. We ignored the alcohol group on the trialdehyde to avoid asymmetrical cages, assuming it will not greatly impact geometrical stability, leading to our model being isostructural to "CC20".

Parallel edges were filtered from the possible graphs. Nine structure attempts were made per cage model using the atomistic geometry optimisation processes. In the structure generation attempts, the *NetworkX* layouts (kamada, spring and spectral) were used based on the atom graph with a scale of 10 Å and with the topology graph with scales 10 and 20 Å. For each graph, the structure (out of those generated from the multiple initial configurations) with the lowest GFN2-xTB energy was then evaluated with the low-cost r2SCAN-3c composite method[S21] in ORCA[S22–S27] to evaluate the effect of using density functional theory. The $E_{\mathrm{b}}$ computed with r2SCAN-3c is reported in the manuscript. The relative energies of the top four candidates, those within 5 kJ $mol^{-1}$ of the minimum, were also examined after geometry optimisation with r2SCAN-3c. Table S3 shows that the geometry optimisation had little impact on the ranking.

**Table S3:** $E_{\mathrm{b}}$ at different levels of theory for the atomistic models generated through unbiased structure prediction. The keys contain identification information regarding the graph id and the step of the process. The bolded row corresponds to the experimentally observed structure.

| key | GFN2-xTB/GFN2-xTB | GFN2-xTB/r2SCAN-3c | r2SCAN-3c/r2SCAN-3c |
|---|---|---|---|
| p1_1_0_2 | 13.25 | 28.1 | |
| p1_2_1_4 | 15.07 | 22.24 | |
| p1_2_4_6 | 4.99 | 5.72 | |
| p1_3_0_4 | 5.2 | 7.97 | |
| p1_3_1_6 | 3.29 | 6.61 | |
| p1_3_3_6 | 1.2 | 1.51 | 1.46 |
| p1_3_9_0 | 9.52 | 19.84 | |
| p1_3_13_7 | 9.82 | 14.85 | |
| p1_3_15_4 | 10.51 | 19.02 | |
| p1_4_0_3 | 6.17 | 9.34 | |
| p1_4_13_6 | 0.0 | 0.05 | 0.08 |
| p1_4_16_8 | 4.8 | 10.95 | |
| p1_4_19_7 | 4.44 | 7.92 | |
| p1_4_21_5 | 10.02 | 18.68 | |
| p1_4_22_4 | 3.84 | 7.86 | |
| p1_4_24_7 | 5.87 | 9.83 | |
| **p1_4_25_6** | **0.25** | **0.0** | **0.0** |
| p1_4_26_5 | 6.36 | 13.64 | |
| p1_4_31_7 | 3.88 | 6.83 | |
| p1_4_37_8 | 8.74 | 16.94 | |
| p1_4_41_5 | 7.94 | 15.92 | |
| p1_4_44_8 | 10.82 | 20.03 | |
| p1_4_51_3 | 5.63 | 8.02 | |
| p1_4_53_6 | 10.97 | 17.14 | |
| p1_4_54_4 | 1.58 | 4.84 | 4.29 |
| p1_4_55_8 | 7.52 | 12.65 | |
| p1_4_59_6 | 8.11 | 11.6 | |
| p1_4_62_5 | 8.19 | 17.1 | |
| p1_4_64_0 | 5.27 | 13.11 | |

Further consideration of the construction approach is crucial to ensure that the diamine-cyclohexane stereochemistry is maintained when constructing arbitrary graph structures, which is usually achieved in *stk* through a multistep, alchemical workflow.[S28] Hence, we also ran structure prediction using an alchemical approach (Figure S2) and found it did not impact the ranking significantly. There are also deviations in the imine direction with respect to the tritropic building block core, which are not constrained in this approach, highlighting another challenge with atomistic modelling of cage structures.

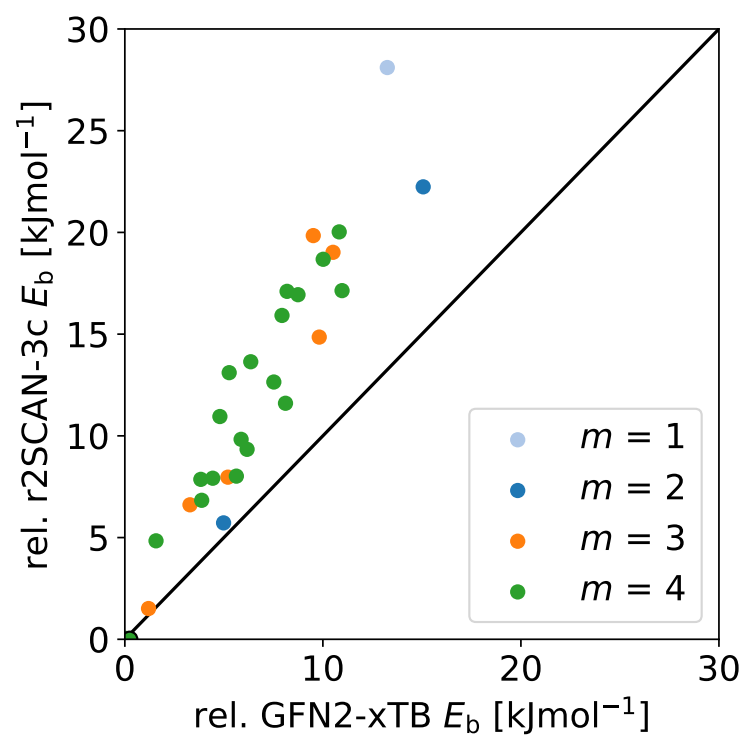


**Fig. S1:** Comparison of the ($x$-axis) GFN2-xTB and ($y$-axis) r2SCAN-3c $E_{\mathrm{b}}$ for the chemical building blocks. The black line is a parity line for comparison.

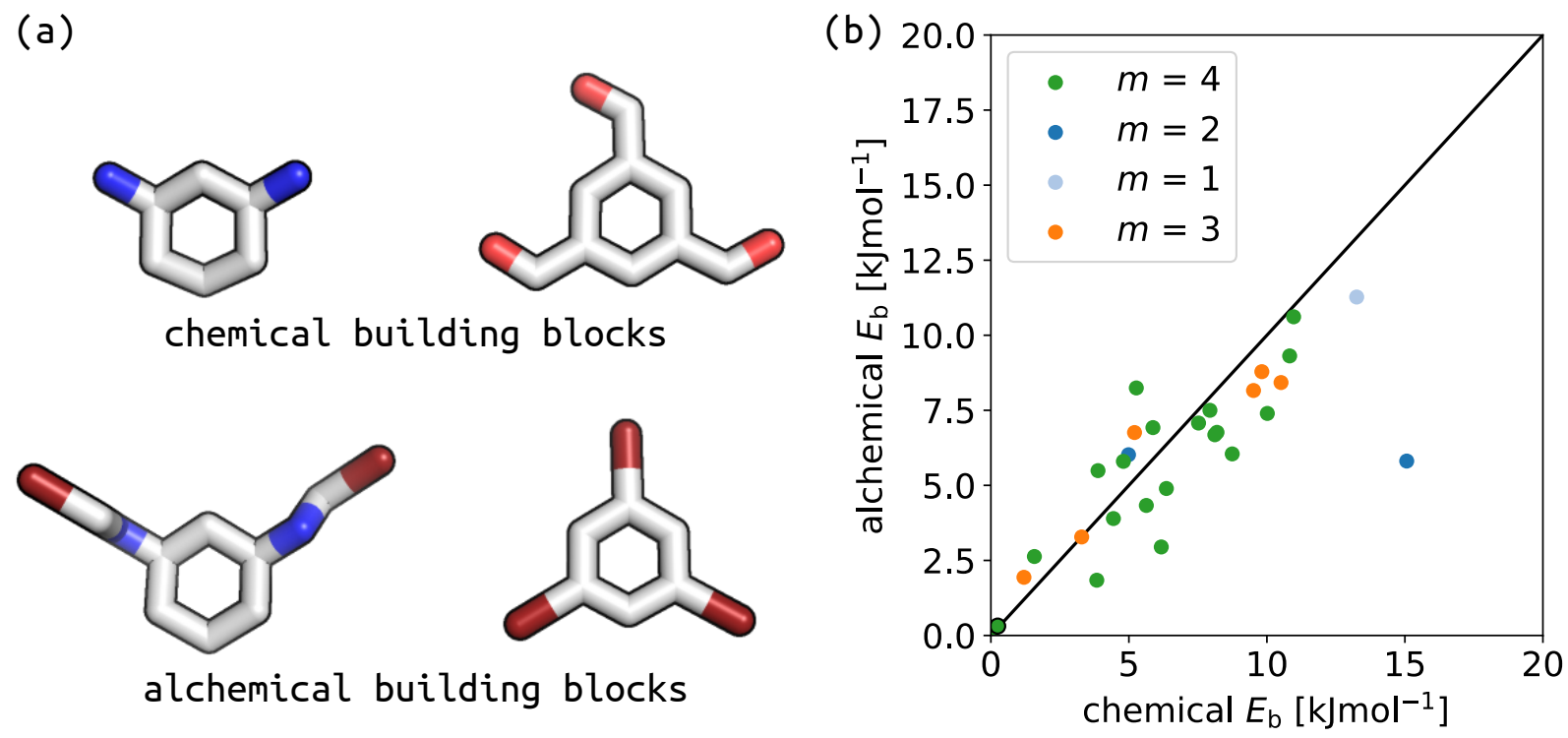


**Fig. S2:** (a) Schematic of the chemical and alchemical construction approach. (b) Comparison of the relative GFN2-xTB energies of all explored structures using ($x$-axis) chemical building blocks and ($y$-axis) alchemical building blocks. The black line is a parity line for comparison.

## S8. Structure prediction validation data

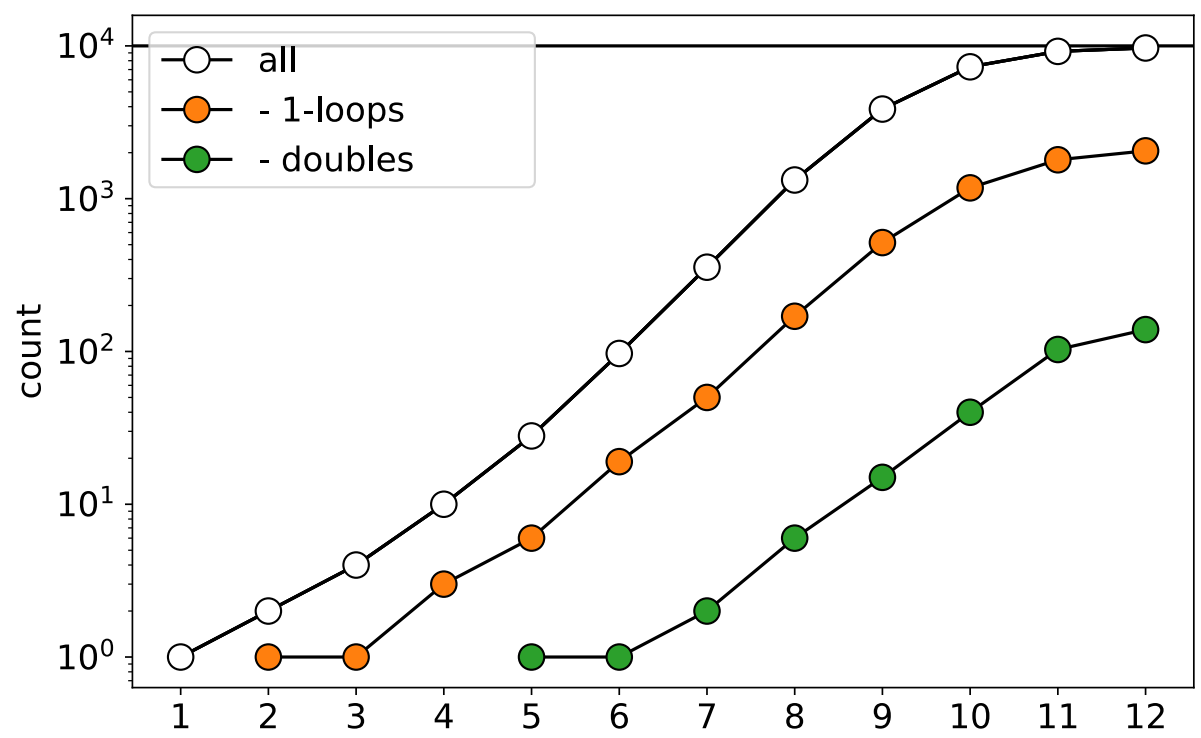


**Fig. S3:** Count of $s = 1:2$, $m = 1-12$ graphs remaining after filtering "parallel edges" (1-loops) and "double-walls", up to a maximum of 10000 for the "all" set.

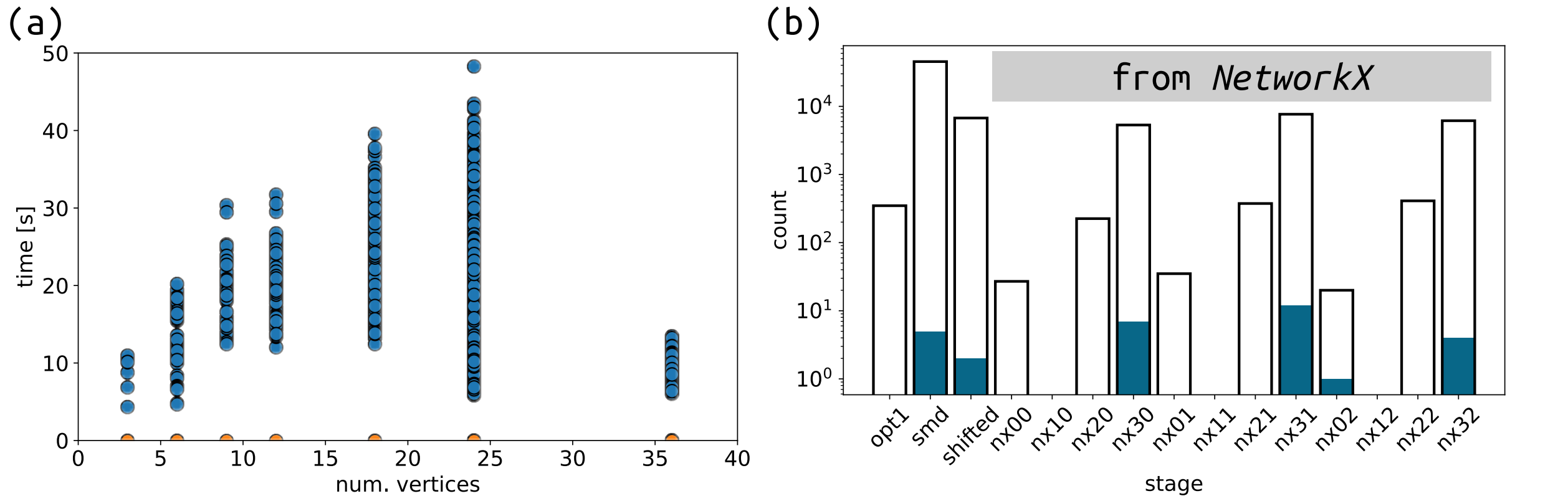


**Fig. S4:** (a) Computation time on 13th Gen Intel i9-13900 (32) CPU with NVIDIA GeForce RTX 4070 GPU for $M_nL_{2n}$ case studies (Section 1.2, prediction data not shown). (b) Count of which geometry optimisation steps produce the minimum energy structure, where *nxij* corresponds to different *NetworkX*[S2] graph layouts and scales as initial configurations for geometry optimisation (**Graphs** steps). *opt1* is the initial geometry optimisation (**Geometry optimisation** step), *smd* represents the soft molecular dynamics (**Soft MD**) step, *shifted* are structures after "shifting" beads and re-optimising the structure (**Shifting** step).

## S9. Mapping atomistic models to minimal models

In this paper, we explore a series of case studies, for which the mapping from atomistic molecules to minimal models is described below. First, we describe the general geometrical considerations going into a minimal-model design.

Square-planar, or metal-containing building blocks are well-defined by their metal coordination, providing a clear underlying bonding geometry. The key feature is the metal-binder bond length, which we use to define a consistent scaling from atomistic to minimal models of 1:2, governed by the approximate Pd-N distance (2 Å). This scaling is used in all systems, including those not considering Pd-N bonding. Throughout, we use the five bead "**cgx.molecular.FourC1Arm**" building block template for tetratopic building blocks. For tritopic building blocks, we use the four bead "**cgx.molecular.ThreeC1Arm**".

We have used two minimal-model ligand models (Figure S5): the rigid three-bead model ("**cgx.molecular.TwoC1Arm**") and hinge-containing, twistable, six-bead minimal model. As in Ref. S29, the mapping to rigid or twistable models comes from the ability of free rotation of the binding sites. Rigid three bead models contain three features defined by minimal-forcefield terms: (i) a binder-angle, (ii) a "ligand size" and (iii) rigidity of rotation through the torsion between binder beads (this is either on or off throughout this paper). Generally, we focus on the distribution of binding-site configurations and extract parameters as close to the binding sites as possible, *i.e.*, determining the ligand size and bite-angle only based on the meta/para-pyridyl configuration.

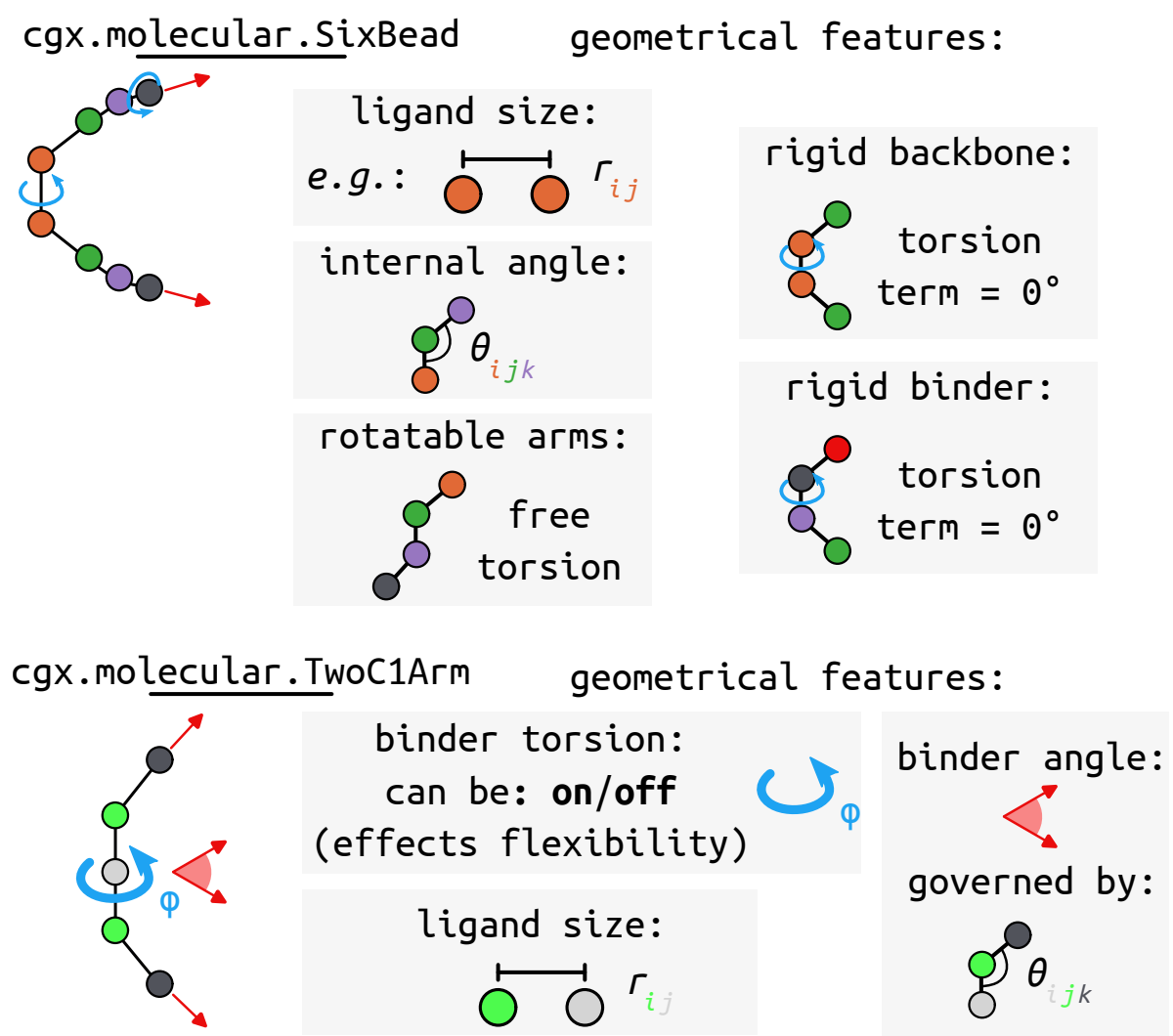


**Fig. S5:** Schematic representation of the two ligand models used in this work and their key geometrical features used to mimic chemical flexibility/rigidity seen at the atomistic level.

Twistable ligands have five geometrical features: (i) ligand size, (ii) internal angle, (iii) rotatable arms, (iv) rigidity of the backbone and (v) rigidity/planarity of the binder interaction. In Ref. S29, for example, these geometric features are well-defined by the rigid pyridyl ring with a bead in the center of the ring and distances/angles defined by a hexagon.

To extract these parameters, a conformer model is needed. In each case, a conformer (either the lowest energy or the conformer most closely resembling the cage conformer) is selected from an ensemble and the measures extracted. Either an ensemble of 100 conformers was generated with RDKit[S5]/ETKDGv3[S18,S19] in bbprepared (https://github.com/andrewtarzia/bbprepared), and a conformer to measure from was selected visually. Or a CREST[S12] conformer search (using the semiempirical GFN2-xTB[S11] for energy evaluation method) was used to produce an ensemble and the conformer with the best alignment of binding sites was chosen for measurement.

## S10. Reproducing "starship" predictions

This case study can be run using script: "`starship_systems.py`"

Six structure attempts were made per cage model with geometry optimisation processes **C** used without *NetworkX* input graphs and **B** with input graphs.

As in Ref. S29, we evaluated structure prediction outcomes for three different values "**cgx.molecular.TwoC1Arm**" ditopic building block (Table S4), exploring different sizes and openings: "L1-s" with a closed model of the rigid ligand, "L1-l" with an open form, "L1b-1" with a smaller binder angle. Seven runs with multiple $s$ and/or $m$ values were performed for each run type, from $m = 1, 2, 3$ and $s = 1 : 1 : 1$, to $m = 1$ and $s = x : y : 2$ or $s = x : y : 3$, where $s = 4 : 2 : 3$ is the experimentally observed outcome and only values of $x$ and $y$ that match the appropriate chemistry were included. Figure S6 shows equivalent results to Ref. S29, although the new algorithm in *cgx* requires significantly less calculations and is more exhaustive.

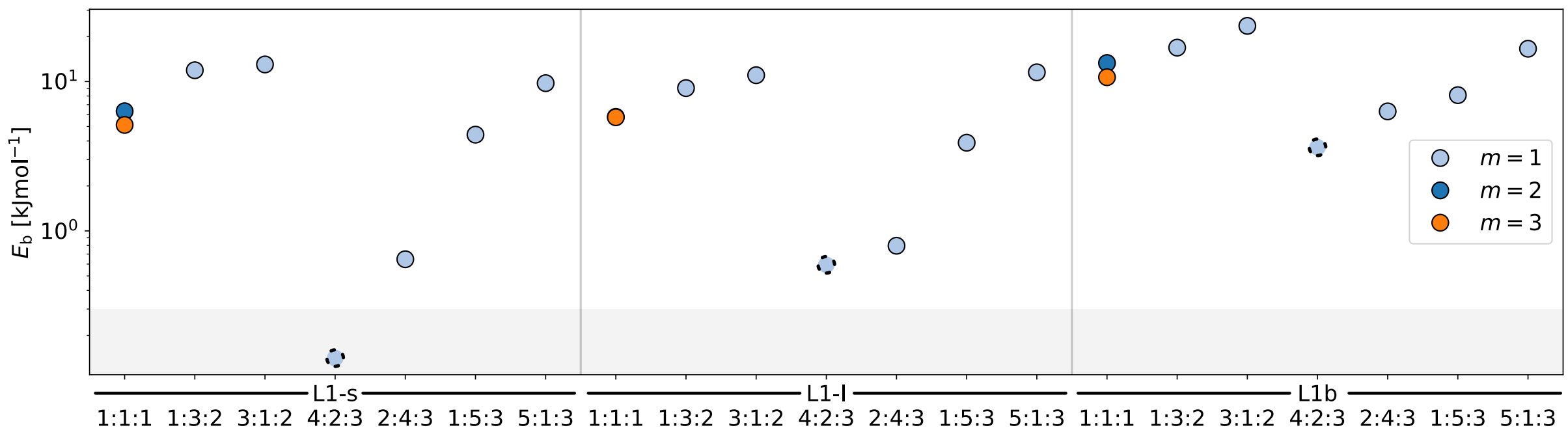


**Fig. S6:** Relative energies of lowest energy structure for a given building block set and $m * s$ combination. Light blue points with a dashed outline correspond to the experimentally observed structure, $m * s = 4 : 2 : 3$, and are lowest in energy, although as the binder angle decreases (in "L1b"), the energy of the starship stoichiometry increases.

**Table S4:** Forcefield terms (specifically equilibrium values, as strength terms were unchanged) used in Section S10.

| | | **runs** | | |
|---|---|---|---|---|
| **term** | **default** | **L1-s** | **L1-l** | **L1b-1** |
| | | bonds [Å] | | |
| *mb* | 1.0 | | | |
| *dd* | 7.0/2 | | | |
| *de* | 1.5/2 | | | |
| *eg* | 1.4/2 | | | |
| *gb* | 1.4/2 | | | |
| *ba* | 2.8/2 | | | |
| *ac* | | 3.9/2/2 | 5.0/2/2 | 3.4/2/2 |
| | | angles [°] | | |
| *bmb* | 90 | | | |
| *dde* | 170 | | | |
| *deg* | 180 | | | |
| *egb* | 120 | | | |
| *mba* | 180 | | | |
| *mbg* | 180 | | | |
| *aca* | 180 | | | |
| *bac* | | 120 | 110 | 90 |
| | | torsions [°] | | |
| *bacab* | 180 | | | |
| *edde* | 180 | | | |
| *mbge* | 180 | | | |
| | | nonbonded [kJ mol$^{-1}$] | | |
| all | 10.0 | | | |

## S11. Forcefield term optimisation from predicted structures

In the function **target_optimisation**, we have implemented a simple, continuous optimisation of the input forcefield parameters of some target structure toward a minimised energy. The databasing in *cgx* parses the forcefield information used to produce the input molecule, and the function takes a set bound (here, maximum changes of 0.5 Å and 20° in bond and angle forcefield terms from the input, respectively, were used) and target terms, providing the optimisation parameters. An optimisation step generates a new forcefield and performs a new geometry optimisation of the input cage geometry with this new forcefield (or the cage geometry from the previous step), and uses the $E_b$ to score the new structure/forcefield. If any parameters become negative, the structure is given a high energy and skipped. The optimisation process uses "duel annealing" in *scipy* with the BFGS method and a tolerance of 0.01, and is run for a maximum of 10 iterations and 400 function calls.[S9]

## S12. Data for "Predicting the structure of ditopic + tritopic porous cages and exploring forcefield modifications"

This case study can be run using script: "`tri_di_systems.py`"

Six structure attempts were made per cage model with geometry optimisation process **C** used without *NetworkX* input graphs and **B** with input graphs.

In this study, we explored the role of length and binder angle in forming **Tri$^2$Di$^3$** or **Tri$^4$Di$^6$** cages.[S30] We explored $s = 2 : 3$ and $m = 1$ and $2$. This study is an interesting challenge because of the difficult-to-map nature of the zirconium cluster and the role of flexibility in the ligand backbone, where the abstraction of our model is more evident. To resolve this issue partly, we initially used manual forcefield manipulations, shown by the array of options in Table S5, where we show the terms of the forcefields applied to access different lengths and ditopic and tritopic building block angles (Figure S7). In particular, torsions were ignored in this study for the linear building blocks (with *bac*= 180°), but turned on, rigidifying the building block, for the bent ("b", with *bac*= 160°) systems. Further development lead to the automatic process in Section S11.

**Table S5:** Forcefield terms (specifically equilibrium values, as strength terms were unchanged) used in Section S12. In this study, there are two terms for each ditopic building block (the "b" term introduces a bend in the building block) and for the tritopic building block ("zr1" vs. "zr2" with altered internal angles. Naming conventions match Ref. S30, as shown in Figure S7.

| | | **runs** | | | | |
|---|---|---|---|---|---|---|
| **term** | **default** | **I1\|I1b - zr1\|zr2** | **I2\|I2b - zr1\|zr2** | **I5\|I5b - zr1\|zr2** | **I6\|I6b - zr1\|zr2** | **I9\|I9b - zr1\|zr2** |
| | | bonds [Å] | | | | |
| *nb* | 3.5/2 | | | | | |
| *ba* | | 3.8/3/2 | 5.7/3/2 | 8.0/3/2 | 9.9/3/2 | 14.2/3/2 |
| *ac* | | 3.8/3/2 | 5.7/3/2 | 8.0/3/2 | 9.9/3/2 | 14.2/3/2 |
| | | angles [°] | | | | |
| *bnb* | | 60\|70 | 60\|70 | 60\|70 | 60\|70 | 60\|70 |
| *nba* | 180 | | | | | |
| *aca* | 180 | | | | | |
| *bac* | | 180\|160 | 180\|160 | 180\|160 | 180\|160 | 180\|160 |
| | | torsions [°] | | | | |
| *bacab* | | -\|180 | -\|180 | -\|180 | -\|180 | -\|180 |
| | | nonbonded [kJ mol$^{-1}$] | | | | |
| all | 10.0 | | | | | |

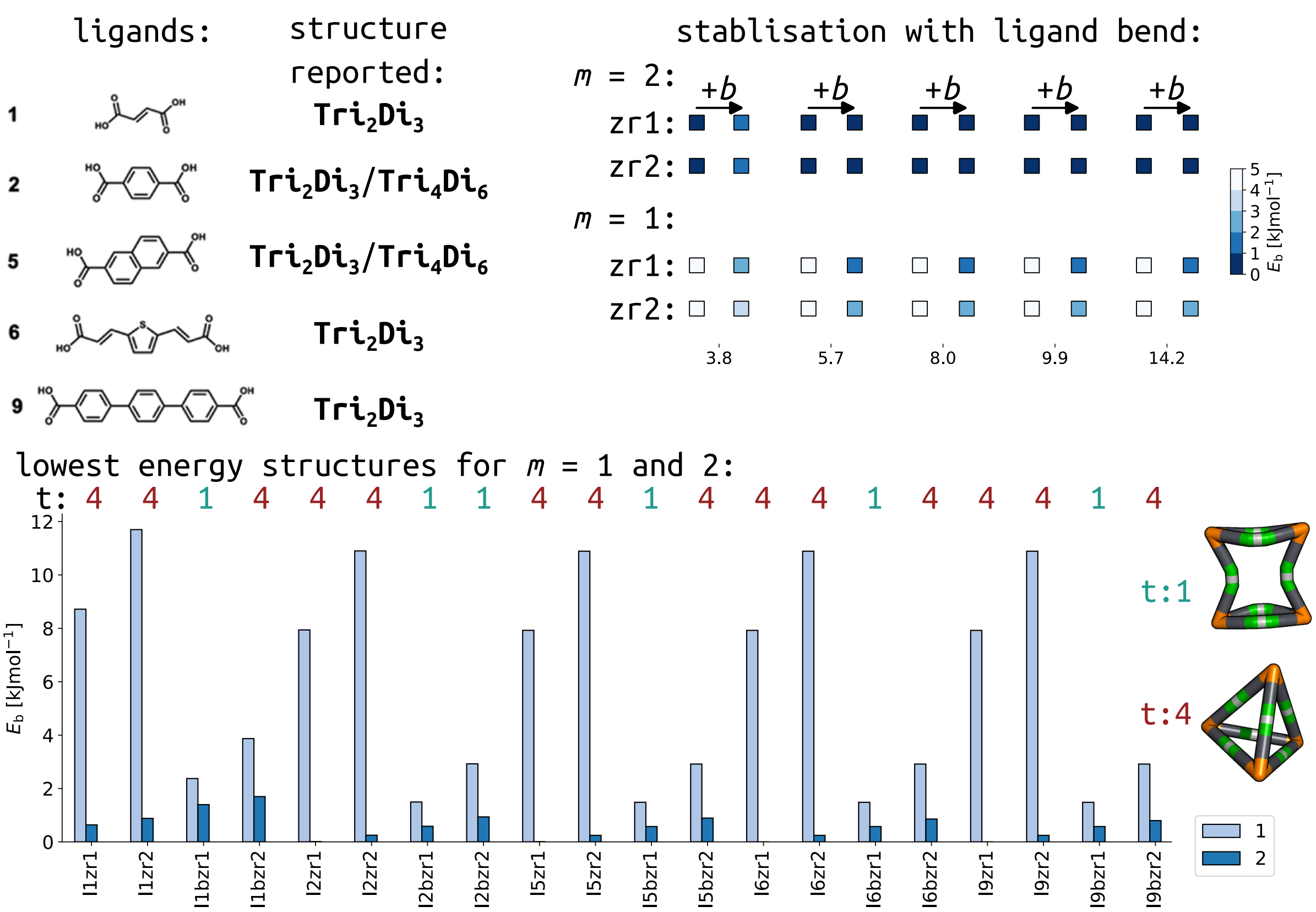


**Fig. S7:** (top left) Ligands studied in this case study and their experimental outcome from Ref. S30. (top right) Comparison of lowest structure energy achieved by changing either tritopic forcefield parameters (vertical) or ditopic forcefield parameters ("+b" corresponds to changing the *bac* angle term from 180° to 160°). (bottom) Lowest energy graph achieved for $m = 1$ and $m = 2$ for all building block and forcefield sets. Example graphs t:1 and t:4, with $m = 2$, shown on the right, where graph t:1 is favoured when the ditopic building block is bent and graph t:4 is the experimentally observed structure.

Through this process (Figure S7), we saw a clear stabilisation of the **Tri$^2$Di$^3$** structure when using the "b" building block

parameters (increasing the bend). However, the $m = 2$ structure is always more stable. Interestingly, adding a bend does stabilise a different graph with $m = 2$ for some runs in Figure S7, leading to flexible tube-like structures[S31] being more stable than the target **Tri$^4$Di$^6$** graph. For all runs, we chose a target graph and building block configuration (Table S6) on which we applied the automatic forcefield optimisation (Section S11). The forcefield optimisation modifies the *nb*, *bnb*, *bac*, *ba* and *ac* terms, with maximum changes of 0.5 Å and 20° from the input bond and angle values, respectively. An example plot of changes is shown in Figure S8, alongside a parity plot of the $E_\mathrm{b}$ before and after forcefield optimisation, showing that most structures are able to achieve near 0 energy, if their range of parameters allows it.

**Table S6:** Target structures for forcefield optimisation.

| runs | structure code | runs | structure code | runs | structure code | runs | structure code |
|---|---|---|---|---|---|---|---|
| **l1 - zr1** | l1zr1_1_0_1 | **l1b - zr1** | l1bzr1_1_0_2 | **l1 - zr2** | l1zr2_1_0_3 | **l1b - zr2** | l1bzr2_1_0_2 |
| **l2 - zr1** | l2zr1_1_0_3 | **l2b - zr1** | l2bzr1_1_0_2 | **l2 - zr2** | l2zr2_1_0_5 | **l2b - zr2** | l2bzr2_1_0_2 |
| **l5 - zr1** | l5zr1_1_0_4 | **l5b - zr1** | l5bzr1_1_0_0 | **l5 - zr2** | l5zr2_1_0_2 | **l5b - zr2** | l5bzr2_1_0_2 |
| **l6 - zr1** | l6zr1_1_0_5 | **l6b - zr1** | l6bzr1_1_0_2 | **l6 - zr2** | l6zr2_1_0_4 | **l6b - zr2** | l6bzr2_1_0_2 |
| **l9 - zr1** | l9zr1_1_0_1 | **l9b - zr1** | l9bzr1_1_0_5 | **l9 - zr2** | l9zr2_1_0_0 | **l9b - zr2** | l9bzr2_1_0_2 |

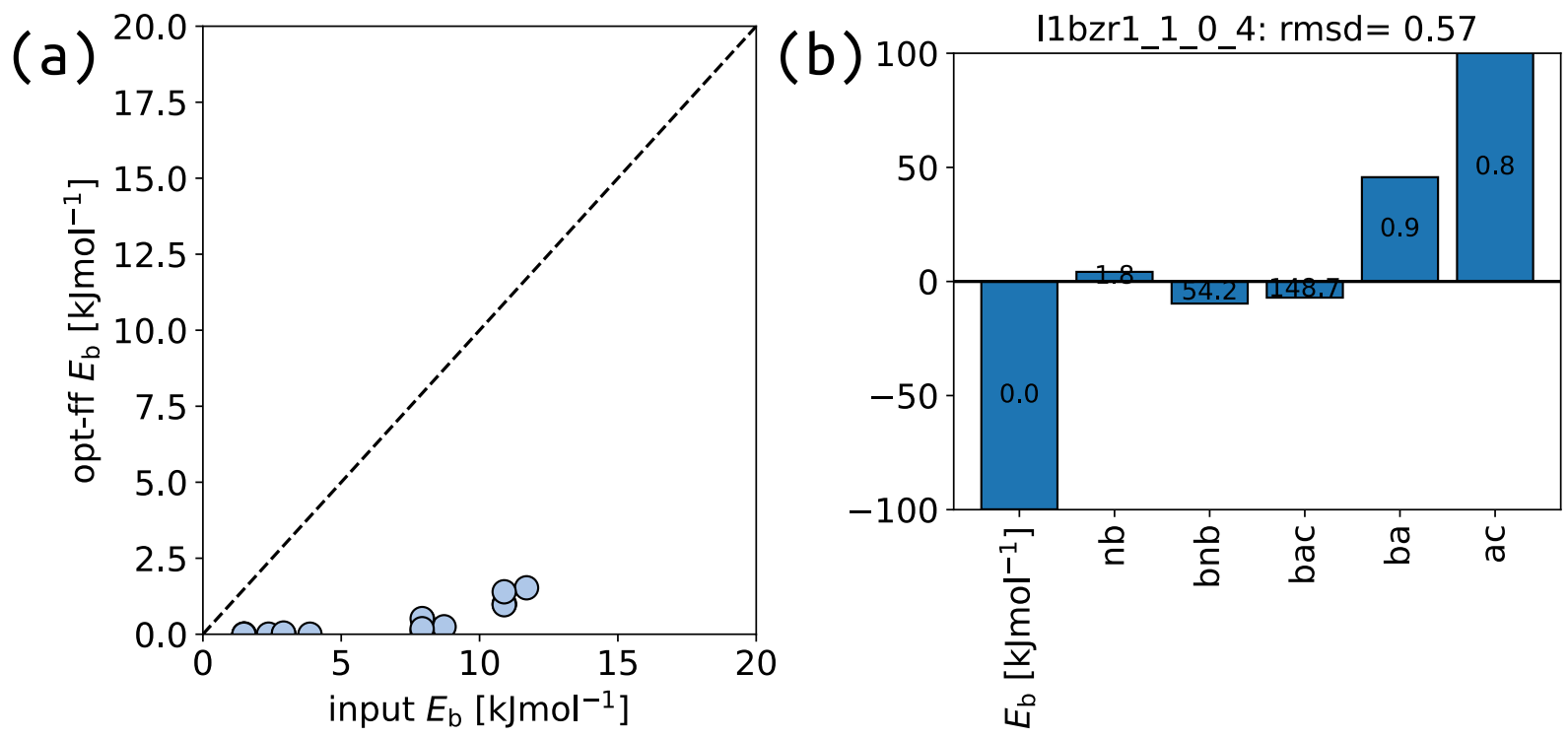


**Fig. S8:** (a) Parity of lowest energy achieved before and after forcefield optimisation for $m = 1$ structures. (b) Percent change in $E_\mathrm{b}$ and optimised input parameters for an example model **Tri$^2$Di$^3$** "l1bzr1_1_0_4". Values shown in bars. The root mean squared deviation (RMSD, in Å) from the generated structure is shown in the title.

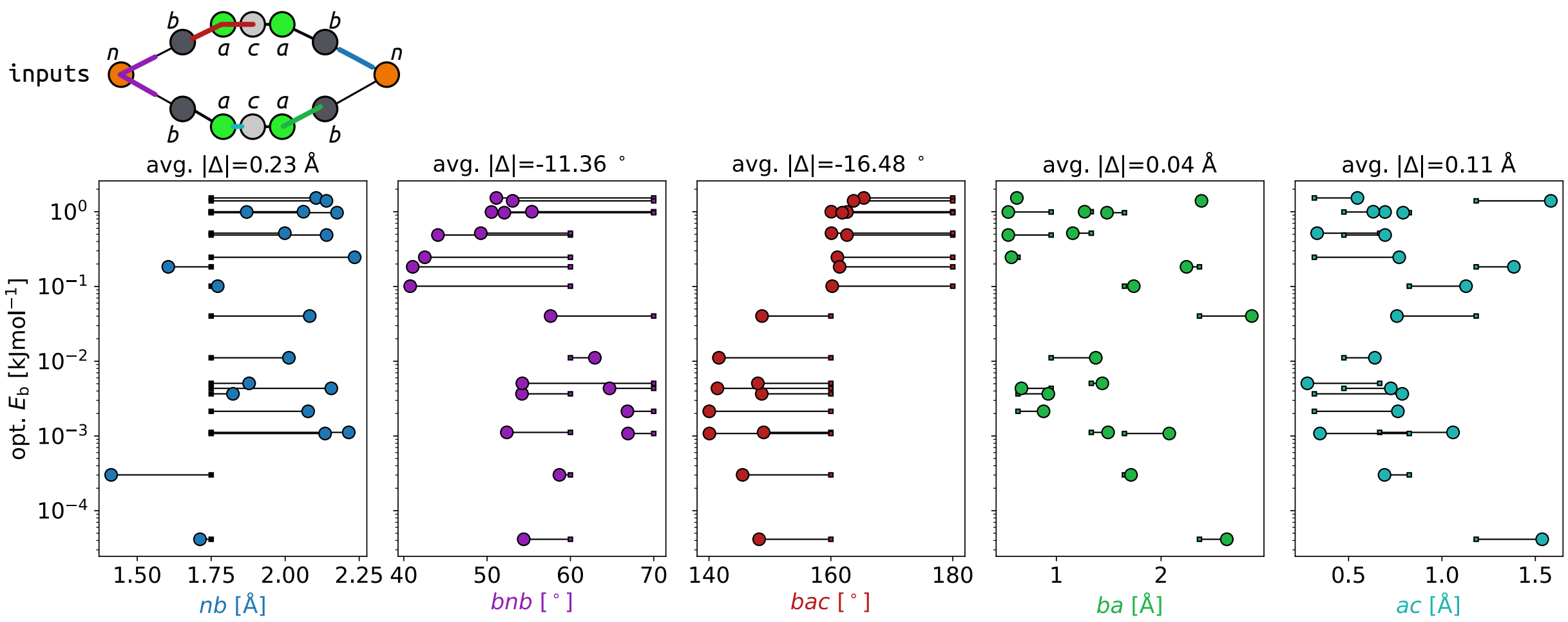


**Fig. S9:** Measuring the effect of this parameter optimisation on the **TwoPlusThree** structures with varying ditopic ligand lengths, showing the change in forcefield term ($x$-axis) and the minimum $E_\mathrm{b}$ achieved in the parameter optimisation. The average change in each term is above each plot. Point colours match the schematic at the top.

## S13. Predicting heteroleptic cage formation of a four component mixture

Lu *et.al.* formed heteroligand structures by mixing Pd(II) (square-planar, tetratopic) with a bidentate building block, a linear building block and a flexible, bent building block (*i.e.*, four building blocks; Figure S10).[S32] In their report, they altered the shape and flexibility of the bent building block by modifying the substitution patterns (Figure S10). To reproduce this, we tested structure prediction over multiple cases with the different linear and bent building block amounts in a standard ratio of **M:bidentate:linear:bent** of 2:2:2-$x$:$x$, where $x$ is 0, 1 or 2, to perform structure prediction of the heteroleptic and homoleptic species.

This case study can be run using script: "`four_comp_systems.py`"

Six structure attempts were made per cage model with geometry optimisation processes **C** used without *NetworkX* input graphs and **A** with input graphs.

In each mixture, there are two square-planar building blocks, and two bidentate cappers with a 90° angle, mixed with combinations of linear or bent ditopic building blocks. For the capper, we introduced a new building block template: `cgx.molecular.TwoC0Arm`, that only has one bead. The forcefield parameters for the five distinct runs ("mix1" to "mix5") are shown in Table S7. The bent building block is quite flexible, and we approximately evaluated three parameter sets for it: "mxy" and "pxy" (measured from the conformer of the meta or para substituted building blocks (Figure S10)), and "fxy", which explores a more open binder angle. We also explored "lin2", which introduces a bend to the linear building block seen in reported crystal structures. Figure S10(b) shows that these changes in parameters do alter the stability of the heteroleptic cage, as expected, but do not decrease the energy sufficiently compared to the homoleptic, bent cage. As we found previously, having no torsional restrictions does result in a significant flattening of the energy landscape.[S14]

**Table S7:** Forcefield terms (specifically equilibrium values, as strength terms were unchanged) used in Section S13. Figure S10 shows the bead mapping to minimal models.

| | | runs | | | | |
|---|---|---|---|---|---|---|
| **term components:** | **default** | **mix1** lin-mxy | **mix2** lin-pxy | **mix3** lin-fxy | **mix4** lin2-mxy | **mix5** lin2-fxy |
| | | bonds [Å] | | | | |
| *mb* | 1.0 | | | | | |
| *ba* | 2.8/2 | | | | | |
| *be* | | 7.6/2 | 7.7/2 | 7.7/2 | 7.6/2 | 7.7/2 |
| *bf* | 2.0/2 | | | | | |
| *ac* | 1.5/2/2 | | | | | |
| *ed* | | 5.0/2/2 | 5.8/2/2 | 5.8/2/2 | 5.0/2/2 | 5.8/2/2 |
| | | angles [°] | | | | |
| *bmb* | 90 | | | | | |
| *mba* | 180 | | | | | |
| *mbe* | 180 | | | | | |
| *bfb* | 90 | | | | | |
| *fbm* | 90 | | | | | |
| *aca* | 180 | | | | | |
| *ede* | 180 | | | | | |
| *bac* | | 180 | 180 | 180 | 175 | 175 |
| *bed* | | 90 | 110 | 120 | 90 | 110 |
| | | torsions [°] | | | | |
| | | nonbonded [kJ mol$^{-1}$] | | | | |
| all | 10.0 | | | | | |

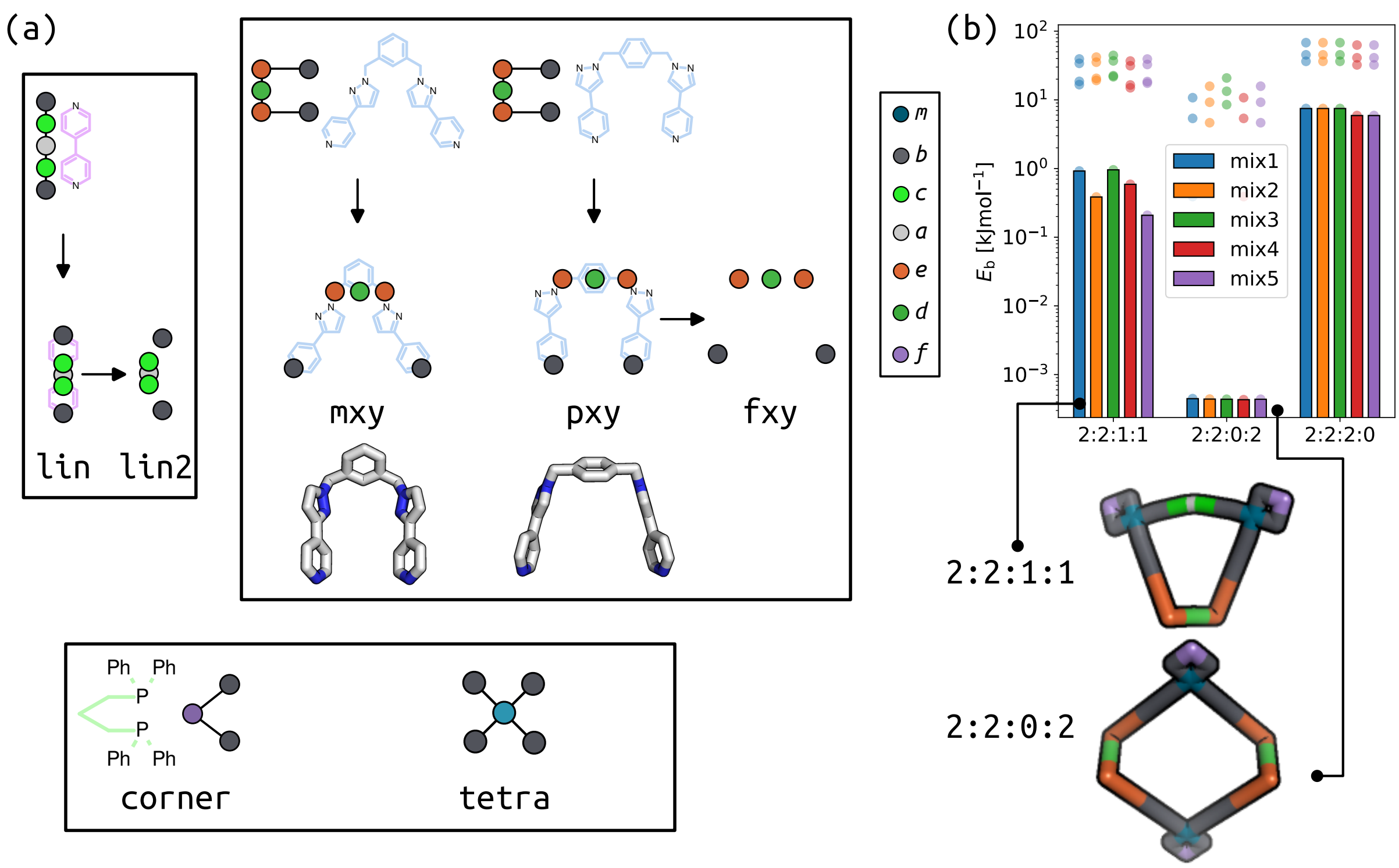


**Fig. S10:** (a) Building block to minimal model mapping in this case study. We show the alterations made to produce each component, and atomistic conformers used to approximate the forcefield terms at the bottom. (b) $E_\mathrm{b}$ of graphs achieved for each run and stoichiometry. The bar shows the minimum energy, while each point is another graph or building block configuration with the same stoichiometry. Structures of stable heteroleptic and homoleptic structures shown at the bottom.

We find that the expected "stirrup" structure is most stable in all heteroleptic cases ($E_\mathrm{b}$ between 1–0.4 kJ mol$^{-1}$), but the

homoleptic structure containing the bent ligand (2:2:0:2) is more stable than these in all cases, while the homoleptic structure containing the linear ligand (2:2:2:0) is less stable (Figure S10). Hence, we do not predict the experimental, heteroleptic outcome to be the energetically most stable. However, the energetic preference for the heteroleptic species over the homoleptic-linear species may favour the formation of the heteroleptic structure when all components are mixed in solution. Additionally, solution analysis (2D-NMR) in the experimental report shows that there is a mixture of complexes formed for system **mXy** ("*mix1*"), although there is no apparent mixture for **pXy** ("*mix2*"), which agrees with the improved strain energy for *mix2* with our model. When visualising the crystal structures, we see that the linear building block can actually bend up to 10° (REFCODE **LUKMAG** has Pd-N-N angle of 166°).[S32] Again the choice of input model parameters (distances, angles), and flexibility is crucial here. Hence, we tested how changes in input binder angles changes the structure prediction outcome (predictions *mix3*, *mix4* and *mix5* in Figure S10). While these changes do stabilise the "stirrup" target structure, it does not alter the trend in structure stability (homoleptic bent > heteroleptic stirrup > homoleptic linear).

## S14. Genetic algorithm (GA) exploration

Throughout, we use a fitness function focussing on minimising $E_\mathrm{b}$ as

$$f = e^{-\beta E_\mathrm{b}}, \qquad \text{[S6]}$$

where $\beta = 5\,\mathrm{mol\,kJ^{-1}}$, giving an exponential decay of $f$ from 1 to 0 within $\sim 2\,\mathrm{kJ\,mol^{-1}}$. In all cases, the cage structures were geometry optimised using method **D** only (Table S2).

The hierarchical GA process is adaptable, and various changes are expected in the future. The current form performs 6 individual GAs (Figure S11) toward a lowest energy structure for a given building block, forcefield, stoichiometry ($s$) and multiplier ($m$) combination. The first four GAs are run concurrently, with a short configuration to initially explore space, which is narrowed down with a long-run configuration in GA5 over the elite population and again in GA6 over neighbours, selecting for a specific graph id (Table S8). There are only two chromosomes: i) the underlying topology graph defining the connectivity of building blocks and ii) the building block configuration, defining where each distinct ditopic building block is placed on the graph. The hierarchical process is ideal for using far fewer calculations to answer questions about broad trends in preferred graph structures.

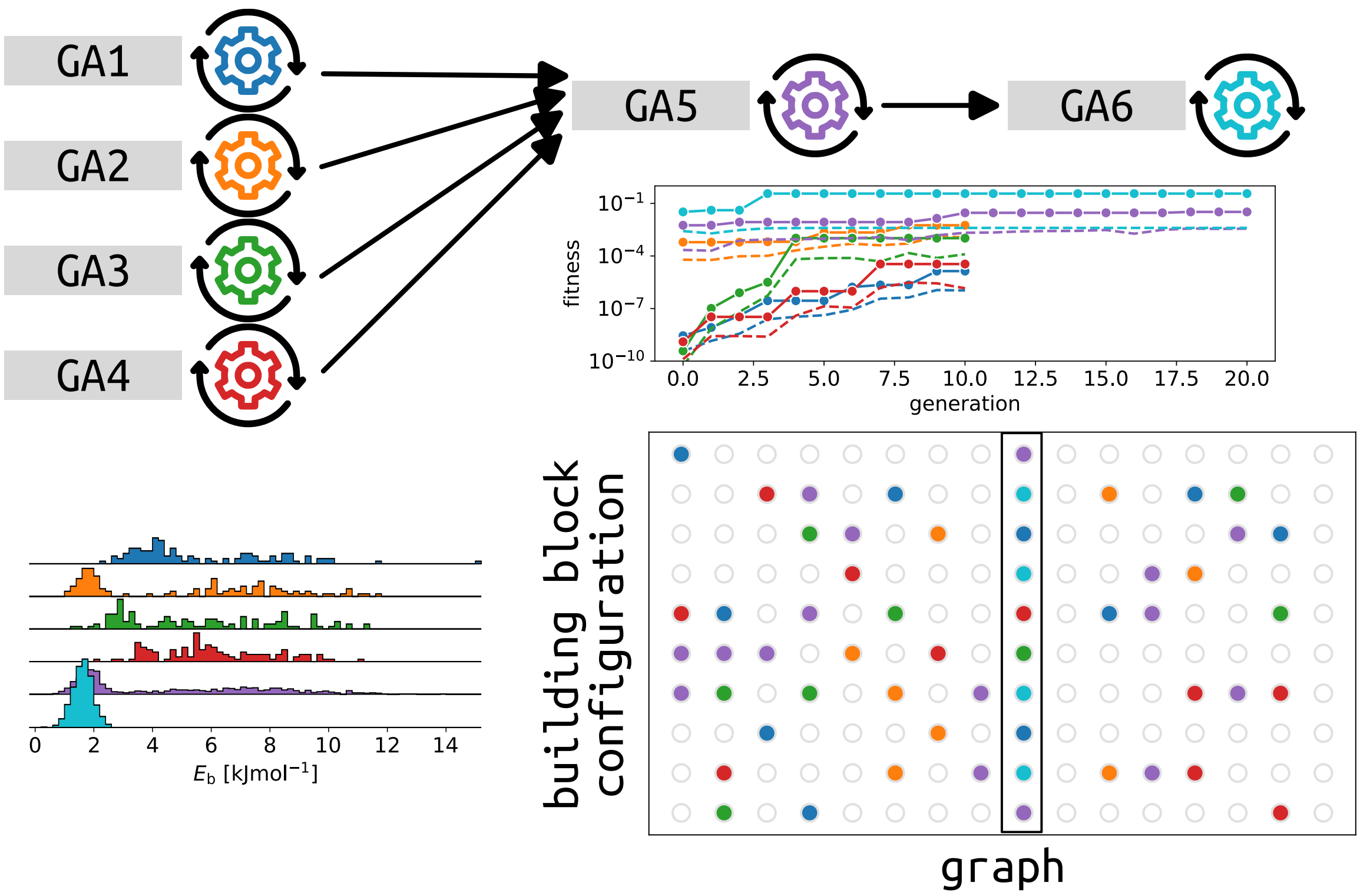


**Fig. S11:** (top) Initially, four short GA runs with random initial configurations are run, the elite population of which are used to initialise GA5. The fitness in these GA runs are shown below, as well as the energy distributions of each (bottom left). (bottom right) Schematic of the two dimensional gene map explored in this process, where the highlighted column shows how a single graph is focussed in on in GA6.

**Table S8:** GA run configurations. Restrictions in GA6 correspond to the graph isolation shown in the inset box in Figure S11 (bottom-right).

| run | GA1 | GA2 | GA3 | GA4 | GA5 | GA6 |
|---|---|---|---|---|---|---|
| seed | 4 | 12689 | 18 | 999 | 142 | 6582 |
| unchanged steps | 2 | 2 | 2 | 2 | 5 | N/A |
| mutations | 2 | 2 | 2 | 2 | 10 | N/A |
| generations | 10 | 10 | 10 | 10 | 20 | 20 |
| selections | 10 | 10 | 10 | 10 | 20 | 200 |
| restrictions | | | | | | graph id |

The general process for a GA run is as follows:

1. A random chromosome selection of **selections** size is used for generation zero.
2. The structure, and then fitness, of each chromosome in a generation is generated/evaluated.
3. A series of chromosomes are added to the current generation through mutations and crossovers. Each process adds **mutations** chromosomes to a new generation list. Random mutations of each chromosome are applied in sequence to add **mutations** for each chromosome type (here, just graph and building block configuration). Similarly, a roulette mutation is applied to each chromosome type. Random and roulette selection is also applied to crossover processes to add **mutations** twice to the new generation based on two known chromosomes. If "**unchanged steps**" generations pass without a change in best chromosome, the number of mutations is doubled for one step.
   - In GA6 (which is strictly not an example of a genetic algorithm), the graph is constant, and no mutations of crossovers are performed. Only the neighbouring chromosomes are considered shifting genes $\pm 4$ indices.
4. The **mutations** best chromosomes from the existing generation are added to the next generation. In GA5 and GA6, the whole known population is added.
5. The generation is deduplicated, and the best **selections** chromosomes move on to the next generation.
6. Steps 3 to 6 are repeated for **generations** cycles.

## S15. Data for "Applying our method to higher nuclearity heteroleptic $Cu_{18}L^AL^B$ cages."

This case study can be run using script: "`cu18_systems.py`"

Only one structure attempt was made in this study, for efficiency, alongside optimisation workflow **D**, which takes advantage of the *NetworkX* layouts (spectral and kamada) throughout.

The hierarchical nature of the genetic algorithm applied here (Section S14) highlights the potential use of adaptive algorithms in structure prediction. For example, an initial test run showed quickly that graphs containing parallel bonds were not worth simulating for these building blocks, so filtering for them in a new run (taking advantage of that initial data) narrowed our search space significantly. Similarly, it is clear early on that certain stoichiometries are not stable, and certain graph configurations are not either, without needing to study them exhaustively.

The list of stoichiometries $(s * m)$ tested was: $1:1:1$ for $m = 1-9$, $1:3:2$, $3:1:2$, $2:4:3$, $4:2:3$, $4:8:6$, $8:4:6$, $6:12:9$ and $12:6:9$. We also explored the effect of input geometry measures, which did not alter significantly the outcome, but did show some changes in the relative energies. The experimental structure was always the most stable. The four different configurations all map to the building block with the 120° bite angle,[S33] or binder angle around 150°, where we explored placing the beads adjacent to the carboxylate or the central benzene ring, as well as changing the binder angle slightly based on measurements of the crystal structure that showed a binder angle closer to 145° (Table S9).

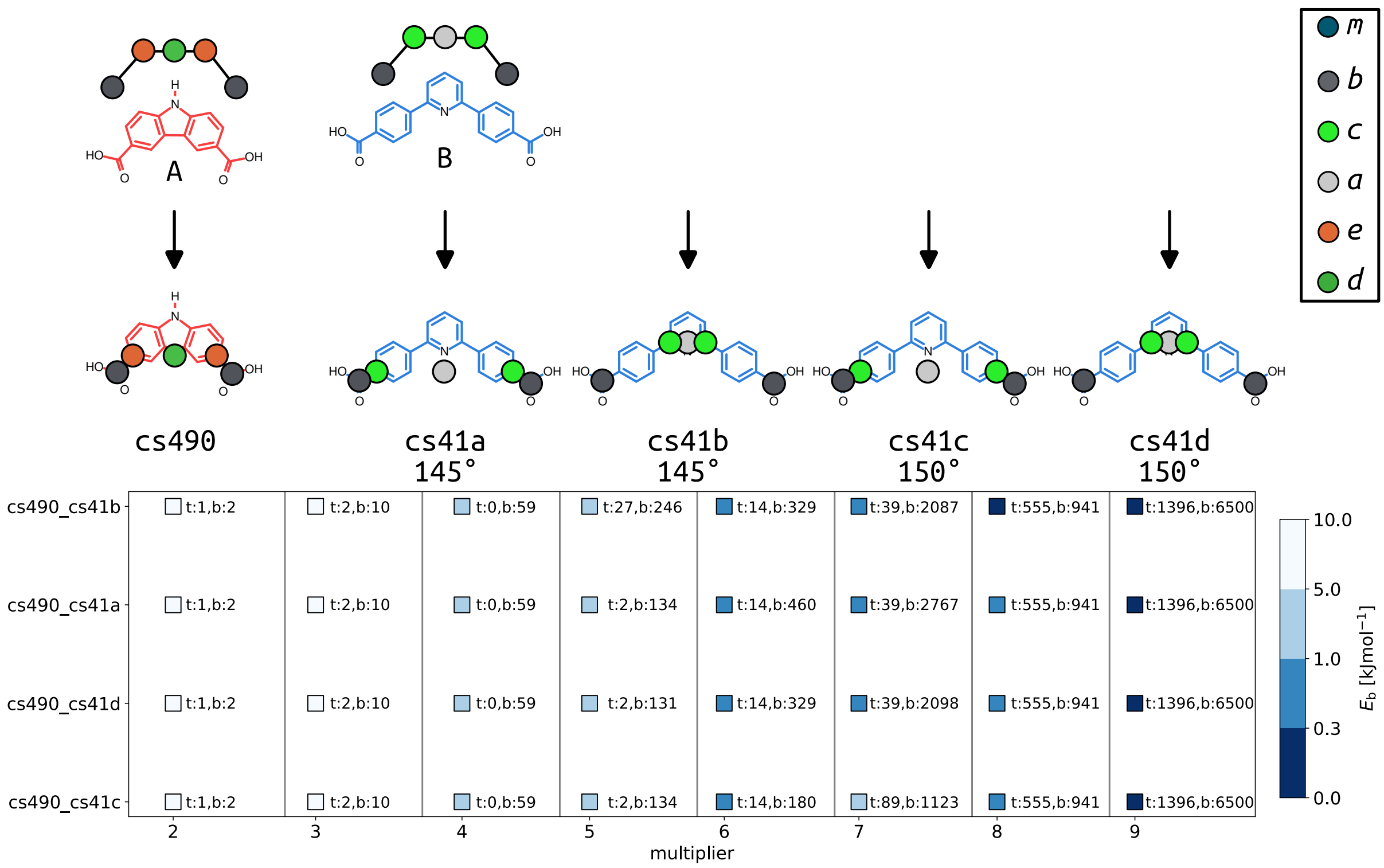


**Fig. S12:** (top) Mapping used for this case study, showing the mapping and forcefield differences for four different initial parameter settings. (bottom) Map of lowest energy structure for each $m$ for the four different runs. Note that each square may include multiple stoichiometries. Graph ("t") and building block configuration ("b") index of the lowest energy structure is shown on the plot.

**Table S9:** Forcefield terms (specifically equilibrium values, as strength terms were unchanged) used in Section S15.

| | runs | | | | |
|---|---|---|---|---|---|
| **term** | **default** | **cs490_cs41a** | **cs490_cs41b** | **cs490_cs41c** | **cs490_cs41d** |
| | | bonds [Å] | | | |
| *mb* | 1.0 | | | | |
| *ba* | | 1.5/2 | 5.7/2 | 1.5/2 | 5.7/2 |
| *be* | 1.5/2 | | | | |
| *ac* | | 9.5/2/2 | 2.4/2/2 | 9.5/2/2 | 2.4/2/2 |
| *ed* | 5.9/2/2 | | | | |
| | | angles [°] | | | |
| *bmb* | 90 | | | | |
| *mba* | 180 | | | | |
| *mbe* | 180 | | | | |
| *aca* | 180 | | | | |
| *ede* | 180 | | | | |
| *bac* | | 145 | 150 | 145 | 150 |
| *bed* | 135 | | | | |
| | | torsions [°] | | | |
| *bacab* | 180 | | | | |
| *bedeb* | 180 | | | | |
| | | nonbonded [kJ $mol^{-1}$] | | | |
| all | 10.0 | | | | |

## References


(S1) Fraux, G.; Cersonsky, R.K.; and Ceriotti, M. “Chemiscope: Interactive Structure-Property Explorer for Materials and Molecules.” *J Open Source Softw*, 2020. **5**(51):2117. doi:10.21105/joss.02117
(S2) Hagberg, A.A.; Schult, D.A.; and Swart, P.J. “Exploring Network Structure, Dynamics, and Function Using NetworkX.” In G. Varoquaux; T. Vaught; and J. Millman (eds.), “Proceedings of the 7th Python in Science Conference,” Pasadena, CA USA, 2008 pp. 11–15
(S3) Harris, C.R.; Millman, K.J.; van der Walt, S.J.; Gommers, R.; Virtanen, P.; Cournapeau, D.; Wieser, E.; Taylor, J.; Berg, S.; Smith, N.J.; Kern, R.; Picus, M.; Hoyer, S.; van Kerkwijk, M.H.; Brett, M.; Haldane, A.; del Río, J.F.; Wiebe, M.; Peterson, P.; Gérard-Marchant, P.; Sheppard, K.; Reddy, T.; Weckesser, W.; Abbasi, H.; Gohlke, C.; and Oliphant, T.E. “Array Programming with NumPy.” *Nature*, 2020. **585**(7825):357–362. doi:10.1038/s41586-020-2649-2
(S4) Eastman, P.; Galvelis, R.; Peláez, R.P.; Abreu, C.R.A.; Farr, S.E.; Gallicchio, E.; Gorenko, A.; Henry, M.M.; Hu, F.; Huang, J.; Krämer, A.; Michel, J.; Mitchell, J.A.; Pande, V.S.; Rodrigues, J.P.; Rodriguez-Guerra, J.; Simmonett, A.C.; Singh, S.; Swails, J.; Turner, P.; Wang, Y.; Zhang, I.; Chodera, J.D.; De Fabritiis, G.; and Markland, T.E. “OpenMM 8: Molecular Dynamics Simulation with Machine Learning Potentials.” *J Phys Chem B*, 2024. **128**(1):109–116. doi:10.1021/acs.jpcb.3c06662
(S5) Landrum, G.; Tosco, P.; Kelley, B.; Rodriguez, R.; Cosgrove, D.; Vianello, R.; sriniker; gedeck; Jones, G.; NadineSchneider; Kawashima, E.; Nealschneider, D.; Dalke, A.; Swain, M.; Cole, B.; Turk, S.; Savelev, A.; Vaucher, A.; Wójcikowski, M.; Take, I.; Scalfani, V.F.; Probst, D.; Ujihara, K.; Walker, R.; Pahl, A.; godin, g.; tadhurst-cdd; Lehtivarjo, J.; Bérenger, F.; and Bisson, J. “Rdkit/Rdkit: 2024_03_4 (Q1 2024) Release.” Zenodo, 2024. doi:10.5281/zenodo.12604375
(S6) “Calculate Root-mean-square Deviation (RMSD) of Two Molecules Using Rotation.” http://github.com/charnley/rmsd, 2023. (version 1.6.4)
(S7) Kabsch, W. “A Solution for the Best Rotation to Relate Two Sets of Vectors.” *Acta Cryst A*, 1976. **32**(5):922–923. doi:10.1107/S0567739476001873
(S8) Treinish, M.; Carvalho, I.; Tsilimigkounakis, G.; and Sá, N. “Rustworkx: A High-Performance Graph Library for Python.” *J Open Source Softw*, 2022. **7**(79):3968. doi:10.21105/joss.03968
(S9) Virtanen, P.; Gommers, R.; Oliphant, T.E.; Haberland, M.; Reddy, T.; Cournapeau, D.; Burovski, E.; Peterson, P.; Weckesser, W.; Bright, J.; van der Walt, S.J.; Brett, M.; Wilson, J.; Millman, K.J.; Mayorov, N.; Nelson, A.R.J.; Jones, E.; Kern, R.; Larson, E.; Carey, C.J.; Polat, İ.; Feng, Y.; Moore, E.W.; VanderPlas, J.; Laxalde, D.; Perktold, J.; Cimrman, R.; Henriksen, I.; Quintero, E.A.; Harris, C.R.; Archibald, A.M.; Ribeiro, A.H.; Pedregosa, F.; and van Mulbregt, P. “SciPy 1.0: Fundamental Algorithms for Scientific Computing in Python.” *Nat Methods*, 2020. **17**(3):261–272. doi:10.1038/s41592-019-0686-2
(S10) Turcani, L.; Tarzia, A.; Szczypiński, F.; and Jelfs, K.E. “Stk: An Extendable Python Framework for Automated Molecular and Supramolecular Structure Assembly and Discovery.” *J Chem Phys*, 2021. **154**(21):214102. doi:10.1063/5.0049708
(S11) Bannwarth, C.; Ehlert, S.; and Grimme, S. “GFN2-xTB—An Accurate and Broadly Parametrized Self-Consistent Tight-Binding Quantum Chemical Method with Multipole Electrostatics and Density-Dependent Dispersion Contributions.” *J Chem Theory Comput*, 2019. **15**(3):1652–1671. doi:10.1021/acs.jctc.8b01176
(S12) Pracht, P.; Grimme, S.; Bannwarth, C.; Bohle, F.; Ehlert, S.; Feldmann, G.; Gorges, J.; Müller, M.; Neudecker, T.; Plett, C.; Spicher, S.; Steinbach, P.; Wesołowski, P.A.; and Zeller, F. “CREST—A Program for the Exploration of Low-Energy Molecular Chemical Space.” *J Chem Phys*, 2024. **160**(11):114110. doi:10.1063/5.0197592
(S13) Santolini, V.; Miklitz, M.; Berardo, E.; and E. Jelfs, K. “Topological Landscapes of Porous Organic Cages.” *Nanoscale*, 2017. **9**(16):5280–5298. doi:10.1039/C7NR00703E
(S14) Tarzia, A.; H. Wolpert, E.; E. Jelfs, K.; and M. Pavan, G. “Systematic Exploration of Accessible Topologies of Cage Molecules via Minimalistic Models.” *Chem Sci*, 2023. **14**(44):12506–12517. doi:10.1039/D3SC03991A
(S15) Boothroyd, S.; Behara, P.K.; Madin, O.C.; Hahn, D.F.; Jang, H.; Gapsys, V.; Wagner, J.R.; Horton, J.T.; Dotson, D.L.; Thompson, M.W.; Maat, J.; Gokey, T.; Wang, L.P.; Cole, D.J.; Gilson, M.K.; Chodera, J.D.; Bayly, C.I.; Shirts, M.R.; and Mobley, D.L. “Development and Benchmarking of Open Force Field 2.0.0: The Sage Small Molecule Force Field.” *J Chem Theory Comput*, 2023. **19**(11):3251–3275. doi:10.1021/acs.jctc.3c00039
(S16) Berardo, E.; Turcani, L.; Miklitz, M.; and Jelfs, K.E. “An Evolutionary Algorithm for the Discovery of Porous Organic Cages.” *Chem Sci*, 2018. **9**(45):8513–8527. doi:10.1039/C8SC03560A
(S17) Tosco, P.; Stiefl, N.; and Landrum, G. “Bringing the MMFF Force Field to the RDKit: Implementation and Validation.” *J Cheminform*, 2014. **6**(1):37. doi:10.1186/s13321-014-0037-3
(S18) Riniker, S. and Landrum, G.A. “Better Informed Distance Geometry: Using What We Know To Improve Conformation Generation.” *J Chem Inf Model*, 2015. **55**(12):2562–2574. doi:10.1021/acs.jcim.5b00654
(S19) Wang, S.; Witek, J.; Landrum, G.A.; and Riniker, S. “Improving Conformer Generation for Small Rings and Macrocycles Based on Distance Geometry and Experimental Torsional-Angle Preferences.” *J Chem Inf Model*, 2020. **60**(4):2044–2058. doi:10.1021/acs.jcim.0c00025
(S20) Teng, B.; Little, M.A.; Hasell, T.; Chong, S.Y.; Jelfs, K.E.; Clowes, R.; Briggs, M.E.; and Cooper, A.I. “Synthesis of a Large, Shape-Flexible, Solvatomorphic Porous Organic Cage.” *Cryst Growth Des*, 2019. **19**(7):3647–3651. doi:10.1021/acs.cgd.8b01761
(S21) Grimme, S.; Hansen, A.; Ehlert, S.; and Mewes, J.M. “r2SCAN-3c: A “Swiss Army Knife” Composite Electronic-Structure Method.” *J Chem Phys*, 2021. **154**(6):064103. doi:10.1063/5.0040021
(S22) Kruse, H. and Grimme, S. “A Geometrical Correction for the Inter- and Intra-Molecular Basis Set Superposition Error in Hartree-Fock and Density Functional Theory Calculations for Large Systems.” *J Chem Phys*, 2012. **136**(15):154101. doi:10.1063/1.3700154
(S23) Neese, F. “The SHARK Integral Generation and Digestion System.” *Journal of Computational Chemistry*, 2023. **44**(3):381–396. doi:10.1002/jcc.26942
(S24) Caldeweyher, E.; Ehlert, S.; Hansen, A.; Neugebauer, H.; Spicher, S.; Bannwarth, C.; and Grimme, S. “A Generally Applicable Atomic-Charge Dependent London Dispersion Correction.” *J Chem Phys*, 2019. **150**(15):154122. doi:10.1063/1.5090222
(S25) Lehtola, S.; Steigemann, C.; Oliveira, M.J.T.; and Marques, M.A.L. “Recent Developments in Libxc — A Comprehensive Library of Functionals for Density Functional Theory.” *SoftwareX*, 2018. **7**:1–5. doi:10.1016/j.softx.2017.11.002
(S26) Caldeweyher, E.; Bannwarth, C.; and Grimme, S. “Extension of the D3 Dispersion Coefficient Model.” *J Chem Phys*, 2017. **147**(3):034112. doi:10.1063/1.4993215
(S27) Neese, F. “An Improvement of the Resolution of the Identity Approximation for the Formation of the Coulomb Matrix.” *Journal of Computational Chemistry*, 2003. **24**(14):1740–1747. doi:10.1002/jcc.10318
(S28) Davies, J.A.; Tarzia, A.; Ronson, T.K.; Auras, F.; Jelfs, K.E.; and Nitschke, J.R. “Tetramine Aspect Ratio and Flexibility Determine Framework Symmetry for Zn8L6 Self-Assembled Structures.” *Angew Chem Int Ed*, 2023. **62**(10):e202217987. doi:10.1002/anie.202217987
(S29) Benchimol, E.; Tessarolo, J.; Tarzia, A.; Lee, H.; Jouaiti, A.; Hosseini, M.W.; Pavan, G.M.; and Clever, G.H. “Adjacent Backbone Interactions Control Self-Sorting of Chiral Heteroleptic Pd3A2B4 Isosceles Triangles and Pd4A4C4 Pseudo-Tetrahedra.” *Chem*, 2026. **12**(2). doi:10.1016/j.chempr.2025.102780
(S30) Gosselin, A.J.; Decker, G.E.; McNichols, B.W.; Baumann, J.E.; Yap, G.P.A.; Sellinger, A.; and Bloch, E.D. “Ligand-Based Phase Control in Porous Zirconium Coordination Cages.” *Chem Mater*, 2020. **32**(13):5872–5878. doi:10.1021/acs.chemmater.0c01965
(S31) Hema, K.; Grommet, A.B.; Białek, M.J.; Wang, J.; Schneider, L.; Drechsler, C.; Yanshyna, O.; Diskin-Posner, Y.; Clever, G.H.; and Klajn, R. “Guest Encapsulation Alters the Thermodynamic Landscape of a Coordination Host.” *J Am Chem Soc*, 2023. **145**(45):24755–24764. doi:10.1021/jacs.3c08666
(S32) Lu, J.; Turner, D.R.; Harding, L.P.; and Batten, S.R. “Heteroligand Molecular “Stirrups” Using Conformationally Flexible Ditopic Pyridyl-Pyrazolyl Ligands.” *Inorg Chem*, 2009. **48**(16):7525–7527. doi:10.1021/ic9010967
(S33) Li, J.R. and Zhou, H.C. “Metal–Organic Hendecahedra Assembled from Dinuclear Paddlewheel Nodes and Mixtures of Ditopic Linkers with 120 and 90° Bend Angles.” *Angew Chem Int Ed*, 2009. **48**(45):8465–8468. doi:10.1002/anie.200904722
